# Impact of Sn Lewis Acid Sites on the Dehydration of Cyclohexanol

**DOI:** 10.1021/acscatal.4c01608

**Published:** 2024-07-24

**Authors:** Karen A. Resende, Ruixue Zhao, Yue Liu, Eszter Baráth, Johannes A. Lercher

**Affiliations:** † Department of Chemistry and Catalysis Research Center, Technische Universität München, Lichtenbergstrasse 4, Garching 85748, Germany; ‡ Institute for Integrated Catalysis, Pacific Northwest National Laboratory, Richland, Washington 99354, United States

**Keywords:** Sn/Al ratio, MFI zeolite, dehydration, secondary alcohol, Lewis acid, Bro̷nsted acid

## Abstract

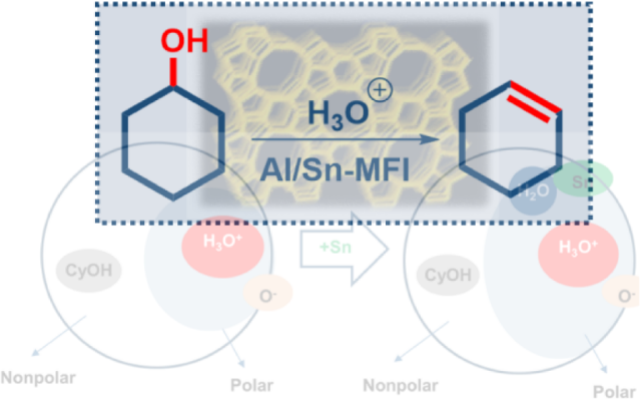

The impact of Sn on the concentration and strength of acid sites in Al containing zeolites with MFI topology and their catalytic activity for the dehydration of cyclohexanol in the aqueous phase has been investigated. The materials maintain constant Al concentrations and consequently Bro̷nsted acid site (BAS) concentrations, while exhibiting an increasing concentration of Sn Lewis acid sites (LAS). The presence of water alters LAS_(Sn)_, leading to weak BAS_(Sn)_ that increases the concentration of water in the zeolite micropore, while leaving the rate of dehydration of cyclohexanol unchanged. The TOF increases with the concentration of BAS_(Al)_ in close contact with framework LAS_(Sn)_, referred to as BAS_(Pair)_. The increase in the Arrhenius pre-exponential factor, without affecting the activation barrier (*E*_a_), leads to the hypothesis that the proximity of both sites allows for a later transition state induced by the polarization of the C–O bond, leading in turn to a higher transition entropy.

## 1 Introduction

Abundant concentrations of water in equilibrium with a Bro̷nsted acidic zeolite converts the hydroxyl groups associated with aluminum substituted into the lattice to hydrated hydronium ions (H_3_O^+^(H_2_O)_*n*_).^1,2^ Because of the higher entropy loss in the pores than in the liquid phase, the size of these clusters is limited. The H_3_O^+^(H_2_O)_*n*_ at lattice aluminum sites are fluxional, but strongly hydrogen bonded clusters. With limited concentrations of aluminum, the limitations of the cluster size (typically 8 water molecules are associated with one proton in the studied MFI) lead to empty voids in the zeolite pores. Organic molecules adsorbed in these pores experience a marked change in their thermodynamic properties, such as being dissolved in a highly ionic solvent. This typically leads to a higher excess chemical potentials of the sorbed molecule, and happens in consequence to a higher reactivity and a weaker interaction with the zeolite.^2^ Together, this may cause higher catalytic activity if the transition state is charged, although the constraints of the zeolite pores induce a decreasing impact at higher H_3_O^+^(H_2_O)_*n*_ concentrations.^2,3^

Unlike the protons of acids, metal cations are structure breakers of such hydrogen bonded networks, forming typically strong direct bonds between the metal cation and water molecules in the first hydration shell.^2^ The presence of hydronium ions and metal cations within a zeolite pore should lead, therefore, to an interesting interplay between the structure-forming protons and structure-breaking metal cations. Hydrolysis of exchangeable cations could, however, cause hydrolysis and may eventually lead to the removal or leaching of (at least a sizable fraction of) these metal cations. Thus, to study such an effect, the metal cations are preferably anchored to the zeolite lattice while remaining accessible to sorbing molecules. The presence of such cations will also influence the polarity of the pore.^3^ Cations like Ti, Sn, and V introduce Lewis acid sites (LAS) into zeolite structures, the specific form depending upon the concentration of water in the pores.^4−7^

Tin substituted in Sn-Beta zeolites, for example, is present in two distinct configurations:^5^ (i) isomorphous substituted Sn^4+^ (close Sn sites), replacing framework Si^4+^ and (ii) hydrolyzed, open Sn sites. Both configurations have specific interactions with water and moderate Lewis acidity; their state can be differentiated via organic probe molecules such as CD_3_CN.^8^ The absence of water in parts of the pore is claimed to aid stabilizing Sn^4+^ Lewis acid sites in several applications.^8−10^

Because of its relatively large cation size and notable Lewis acid strength, we have chosen to use Sn^4+^ as a Lewis acid site to investigate the impact of the combination of H_3_O^+^(H_2_O)_*n*_ and the hydrogen bond breaking action of Sn^4+^. Both the self-organization of water in the pores with varying concentrations of these two sites and the consequences on sorption and catalysis are investigated. The sorption and dehydration of cyclohexanol^2,11,12^ in aqueous phase has been chosen as model case. Previous studies have shown that the rate and elimination mechanism (E1 and E2 routes) depend on the local environment in the zeolite and the bulkiness of the reacting molecule.^12 ,13^ To facilitate interpretation of the mutual impact, a series of Sn-H-ZSM5 (Sn-H-MFI) was synthesized, keeping the aluminum concentration constant and varying the concentrations of Sn. Detailed physicochemical characterization and the impact on cyclohexanol (CyOH) sorption and conversion are used to probe the mutual influence of Bro̷nsted and Lewis acid sites.

## 2 Results and Discussion

### 2.1 Structure of the Zeolites

The normalized powder XRD patterns of the zeolite samples show that at low Sn concentrations, the samples had a typical MFI structure (Table S1) with the main peaks at 2θ = 7.8°, 8.7°, 23.1°, 23.8°, and 24.3° (Figure S1a). For Al/Sn-0.7 crystallization did not occur, indicating an upper limit of Sn incorporation. Diffraction peaks of SnO_2_ (2θ = 31.7°, 36.8°, 41.4°, and 53.1°) were not observed in any sample. A more detailed analysis in the 2θ = 22°–25° range (Figure S1b) suggests somewhat less defined peaks in Sn-MFI and Sn/Al-1. The relative degree of crystallinity calculated for Sn/Al-1 was around 50–60%, while the other samples had values around 100%, when compared with the ZSM-5. The apparent lower crystallinity of Sn/Al-1 is attributed to the generation of defects in zeolite framework.^14^

**Figure 1:**
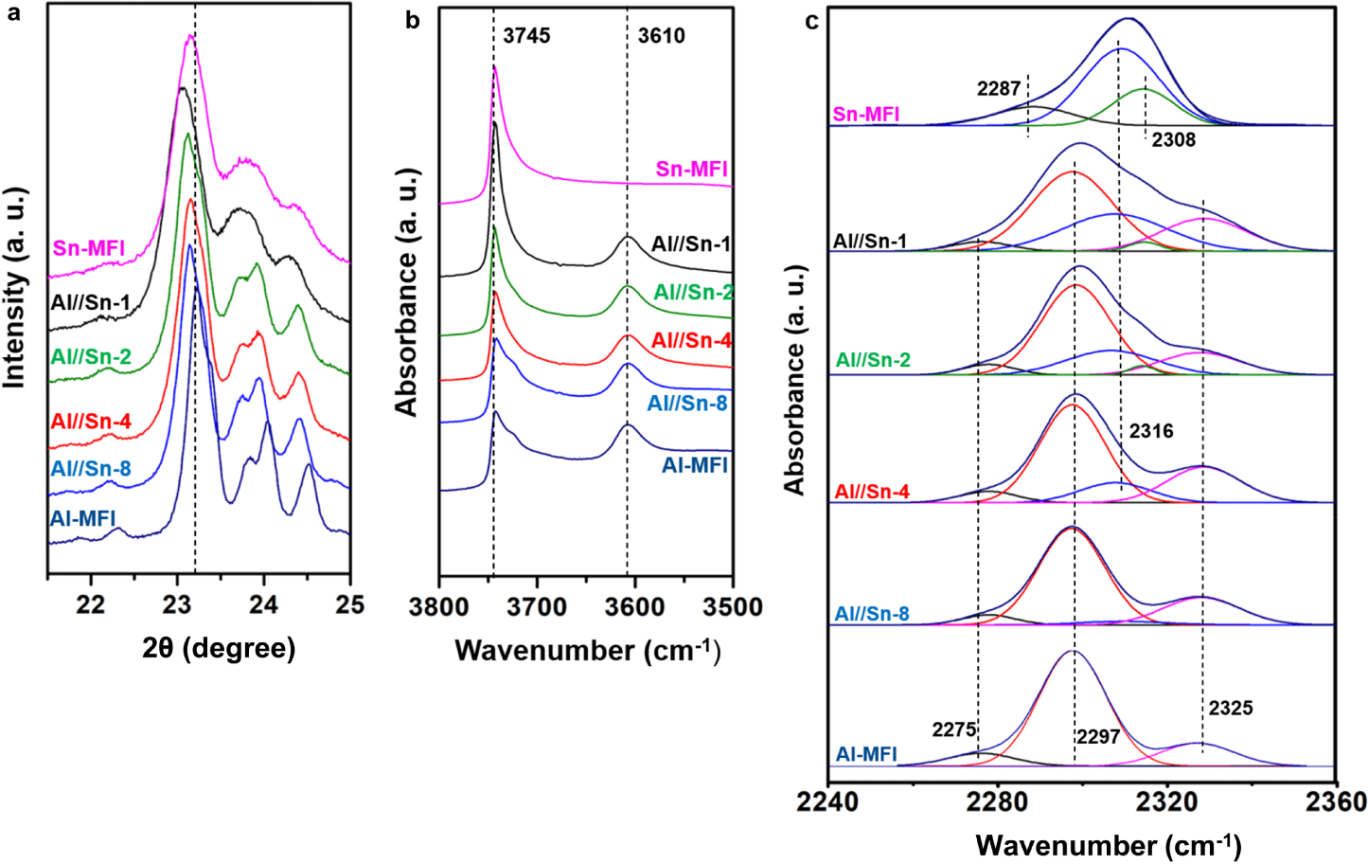
(a) XRD patterns of Al/Sn MFI zeolites showing a specific 2θ range (λ = 1.6531 Å). (b) IR spectra of pyridine adsorption on Al/Sn MFI zeolites. (c) IR spectra of deuterated acetonitrile on Al/Sn MFI zeolites. The deconvolution of the IR spectra was done with the ORIGIN software program using a mixed Gaussian–Lorentzian curve. The bands of adsorbed CD_3_CN were found at 2325 cm^–1^ (magenta) and 2297 cm^–1^ (red). The band at 2287–2275 cm^–1^ (black) is assigned to CD_3_CN adsorbed on silanol groups.

As the substitution leads to adjustment of bond lengths and angles (indicated by shifts of the diffraction peaks)^15^ gradually lower 2θ values (Figure 1a) were observed. The calculated unit cell volume, the crystal system, and the atomic lattice (a–c) are summarized in Table S2. All lattice parameters and the cell volume increased with increasing Sn concentration. This expansion of the orthorhombic unit cell volume as a function of the Sn concentration is consistent with the replacement of Si by Sn, as the bond length of Si–O (0.1623 nm)^16^ is smaller than the bond length of Sn–O (0.2072 nm).

The micropore volumes were <0.2 cm^3^ g^–1^ for all samples (Table 1), in good agreement with the MFI structure. For Al/Sn-8 and Al/Sn-4, the micropore volume slightly increased with the addition of Sn, while for higher concentrations of Sn, the micropore volume decreased, which is hypothesized to be associated with a gradual loss of crystallinity.

**Table 1:** Structural and Acid Site Characterization (for Chemical Composition Please see Table S1)

		pyridine (mmol g^–1^)		CD_3_CN (mmol g^–1^)			
samples	pore volume (cm^3^ g^–1^)	BAS_(Al)_	LAS	LAS_(Sn)_ (2308 cm^–1^)	LAS_(Sn)_ (2316 cm^–1^)	LAS_(EFAL)_ (2325 cm^–1^)	BAS_(Al)_ (2296 cm^–1^)
Sn-MFI	0.14	9.80 × 10^–3^	0.33	0.17	0.13	0	0
Al/Sn-1	0.13	0.21	0.27	0.16	0.02	0.06	0.21
Al/Sn-2	0.16	0.21	0.17	0.09	0.02	0.04	0.22
Al/Sn-4	0.19	0.21	0.13	0.06	-	0.06	0.22
Al/Sn-8	0.18	0.21	0.07	0.01	-	0.05	0.21
Al-MFI	0.17	0.22	0.05	-	-	0.03	0.21

The same tendency was observed for the BET specific surface area values (Table S3). The specific surface area in the mesopore range increased with the addition of Sn.

The infrared spectra (IR) of all the samples showed the OH bands characteristic of ZSM-5 zeolites (Figure S2).^17^ The band at 3745 cm^–1^ is related to silanol groups on the external surface of the zeolites (Figure 1b), while the band at 3610 cm^–1^ is attributed to Bro̷nsted acid sites (BAS), Si(OH)Al.^18^ In general, all the samples showed similar intensities of the band at 3610 cm^–1^, while that at 3745 cm^–1^ increased in the presence of Sn. This suggest that the addition of Sn creates some lattice defects. The Raman spectra of the samples did not show bands around 670 and 560 cm^–1^ (Figure S3), which indicates the absence of extra-framework Sn species.^15,19^

### 2.2 Quantification of Acid Sites

The adsorption of pyridine on Lewis acid sites (LAS) led to IR bands at 1453 and 1490 cm^–1^, while protonation at Bro̷nsted acid sites caused bands at 1490 and 1546 cm^–1^.^8^ The peak assigned to Lewis acid site bound pyridine (1450 cm^–1^) increased with Sn concentration, while the band at 1546 cm^–1^ (BAS) remained constant (Figure S4). For Sn-MFI the 1546 cm^–1^ band was absent, indicating in turn the absence of BAS_(Sn)_ for this sample. Lewis acid sites were observed also for Al-MFI, which is related to extra-framework aluminum (LAS_(EFAl)_). The total concentration of LAS and BAS on each sample was calculated considering equimolar pyridine binding (Table 1).

As expected, all the samples with Al showed a similar concentration of BAS_(Al)_, while the concentration of LAS increased with increasing concentration of Sn^4+^.

The IR spectra after saturation with CD_3_CN and subsequent evacuation at 40 °C are shown in Figure 1c. Adsorption of CD_3_CN allowed to differentiate the acid sites.^20^ The bands at 2316 cm^–1^ (blue) and 2308 cm^–1^ (green) are associated with the C≡N stretching vibrations of CD_3_CN adsorbed on the Sn LAS.^8,21,22^ The bands have been attributed to partially hydrolyzed framework Sn sites (—Si–O—)_3_Sn–OH (open Sn) and fully framework coordinated Sn atoms Sn(—Si–O—)_4_ (close Sn), respectively.^5^ The presence of Al in these samples also leads to LAS associated with extra-framework Al and BAS_(Al)_ associated with framework Al. The bands of adsorbed CD_3_CN on these sites were observed at 2325 cm^–1^ (magenta) and 2297 cm^–1^ (red), respectively (Figure 1). The band at 2287–2275 cm^–1^ (black) is attributed to CD_3_CN adsorbed on silanol groups.^8^ In line with the chemical composition, the concentrations of BAS_(Al)_ and extra-framework Al were nearly identical with all samples, while the concentration of Sn varied (Table 1). As bands characteristic of CD_3_CN on extra-framework Sn were not observed,^23^ we conclude that all the Sn is substituted in the framework, acting as Lewis acid sites (LAS_(Sn)_). For samples with a lower concentration of LAS_(Sn)_, only coordinatively closed tin atoms Sn(—Si–O—)_4_ were observed. The concentration of the open Sn framework centers (—Si–O—)_3_Sn–OH increased with the concentration of Sn. In general, the total concentration of Lewis acid sites (open Sn, close Sn, and extra-framework Al) determined by CD_3_CN was similar to the concentration of Lewis acid sites determined by the adsorption of pyridine.

### 2.3 Interaction with Water and Domain Formation

As Sn introduces additional LAS, it raises questions about how Sn influences the formation and size of hydrated hydronium ions formed from BAS. The concentration of Al in the zeolite framework defines the concentration of the H_3_O^+^(H_2_O)_*n*_.^2^ For Sn-MFI, 4–5 water molecules were adsorbed per Sn (Figure S5).

For aluminum-containing zeolites, the adsorption enthalpy for the first water molecule was 64 kJ mol^–1^, increasing to 82 kJ mol^–1^ with the next water molecule. This was followed by a decrease of the adsorption enthalpy to the enthalpy of water condensation heat (∼45 kJ mol^–1^) at 25 °C as further water molecules adsorbed. A similar behavior was previously observed for H-MFI samples.^2^ For these latter samples, the adsorption enthalpy increase with the second water molecule was associated with proton transfer to adsorbed water, but until now, a conclusive explanation for the observation has not been found.

For activated Sn-MFI the concentration of Bro̷nsted acid sites was very low (∼10 μmol g^–1^). It increased in the presence of water (Figure S6) to approximately 29 μmol g^–1^. The equivalent decrease in the LAS_(Sn)_ was attributed to dissociation of water at the framework Sn sites.^24^

IR spectra after water adsorption at 30 °C on Sn-MFI at 10^–3^–1 bar are compiled in Figure S7. The key bands were observed at 3740, 3648, 3389, and 1615 cm^–1^. The band at 3740 cm^–1^ corresponds to silanol groups located at the external surface. In general, this band was not significantly perturbed by an increase in water pressure, indicating only a very weak adsorption of water. The band at 3648 cm^–1^ is attributed to water adsorbed on accessible Sn sites, analogous to the adsorption of molecular water on Al^3+^. With increasing equilibrium pressure of water, the band of hydrogen bonded water at 3389 cm^–1^ increased in parallel to the band of the deformation vibration of water at 1617 cm^–1^. It is hypothesized that the increase in adsorption enthalpy for Sn-MFI as the concentration of water increased is the consequence of the hydrolysis of the close framework Sn sites to open framework Sn sites and the associated stronger adsorption of water on these sites.^10^

Water adsorption on zeolites substituted with different concentrations of Sn (and similar Al concentrations) (Figure S8) shows a significant increase in the adsorption capacity and strength in comparison to Sn-MFI (Table S4). The total water uptake was defined as the amount of water adsorbed with a heat of adsorption higher than ∼45 kJ/mol (Figure S8). The water uptake on BAS_(Al)_ refers to the amount of water adsorbed on aluminum-containing MFI zeolites (Al-MFI). The additional water uptake increased linearly with the concentration of framework Sn (Figure S9). Averaging overall Sn/Al samples, approximately 4 water molecules were adsorbed per Sn (Table S4). This suggests a stronger interaction of water with incorporated Sn sites than with silanol nests of zeolites.^5,11^

**Figure 2:**
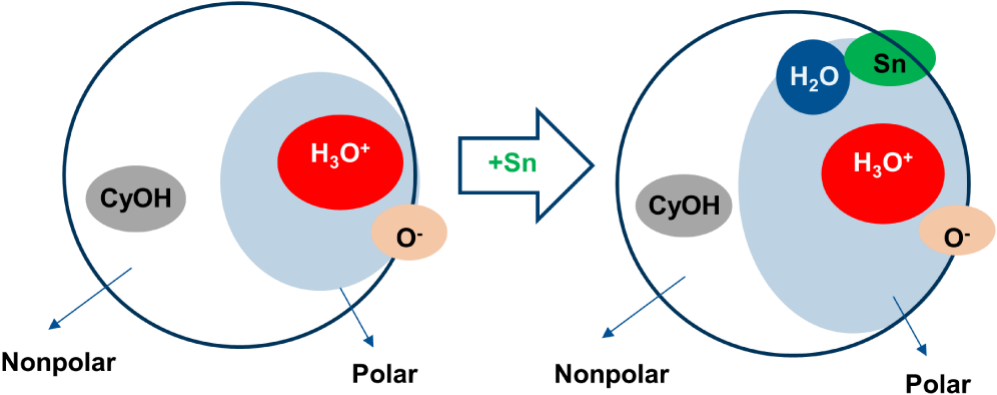
Schematic representation of micropore domains when Sn is present in the zeolite structure. CyOH refers to cyclohexanol molecules inside the nonpolar domain, and O^–^ refers to deprotonated BAS. H_3_O^+^ refers to the hydronium ion, and Sn refers to the framework Sn.

The fraction of accessible space decreased with the concentration of Sn in the framework. Thus, the presence of Sn leads to a higher fraction of the zeolite pores being filled with water (Figure 2). The increased concentration of hydrated hydronium ions resulting from the higher water uptake could potentially influence the distribution and size of proton domains within the zeolite structure. One possibility is that the higher concentration of water leads to more numerous but smaller domains of hydrated hydronium ions distributed throughout the zeolite framework. Alternatively, it could result in fewer but larger domains containing delocalized protons.

To probe this, let us look to the details of adsorption in the zeolite pores in the presence of water. The adsorption isotherm of cyclohexanol in aqueous phase for Al/Sn-2 (Figure S10) showed cyclohexanol uptake to increase sharply with concentration and gradually reaching a constant level above 0.03 mol L^–1^. The uptake of 0.989 mmol g^–1^ corresponds to a volume of sorbed cyclohexanol of 0.104 cm^3^ g^–1^ (Table S5), similar to the volume unoccupied by water (Table S4). The calorimetrically measured adsorption enthalpy (∼40 kJ mol^–1^) was higher than that determined (∼26 kJ mol^–1^) for H-MFI samples.^2^ This high enthalpy of sorption points to a stronger interaction with the sorbed molecule, i.e., a lower excess chemical potential of cyclohexanol compared to cyclohexanol adsorbed on H-MFI with the same concentration of aluminum.

The high enthalpy of sorption is hypothesized to be caused by the stronger and simultaneous interaction of the OH group of cyclohexanol with Sn^4+^ and H_3_O^+^(H_2_O)_*n*_. The requirement of proximity for these sites caused us to denote these sites as paired sites (BAS–LAS_(Pair)_), with BAS and LAS denoting isolated sites. The concentration of BAS–LAS_(Pair)_ in each sample almost entirely corresponds to the concentration of Sn^4+^ (Table 2, for calculations please see Figures S11–S14, Table S6). This observation suggests a strong correlation between the concentration of BAS–LAS_(Pair)_ and the presence of Sn^4+^ ions in the zeolite structure. It indicates that the formation of BAS–LAS_(Pair)_ sites is closely linked to the incorporation of Sn^4+^ ions and Al^3+^ into the zeolite framework. The notable exception for Al/Sn-1, which had around 30% of Sn paired with BAS_(Al)_, is attributed to the lower crystallinity of this sample.

**Table 2:** Catalytic Activity (150 °C, 40 bar) of the MFI Samples

samples	reaction rate × 10^–5^ (mol g_cat_^–1^ s^–1^)	TOF (rate/BAS) (s^–1^)	BAS_(Pair)_/BAS_(total)_	*E*_a_ (kJ mol^–1^)
Sn-MFI	∼0	-	0	-
Al/Sn-2	2.90	0.143	0.469	139 ± 11
Al/Sn-4	2.34	0.118	0.175	145 ± 10
Al/Sn-8	1.91	0.096	0.049	144 ± 7
Al-MFI	1.90	0.087	0	142 ± 7
BAS_(Pair)_	-	0.207	-	140 ± 9

**Figure 3:**
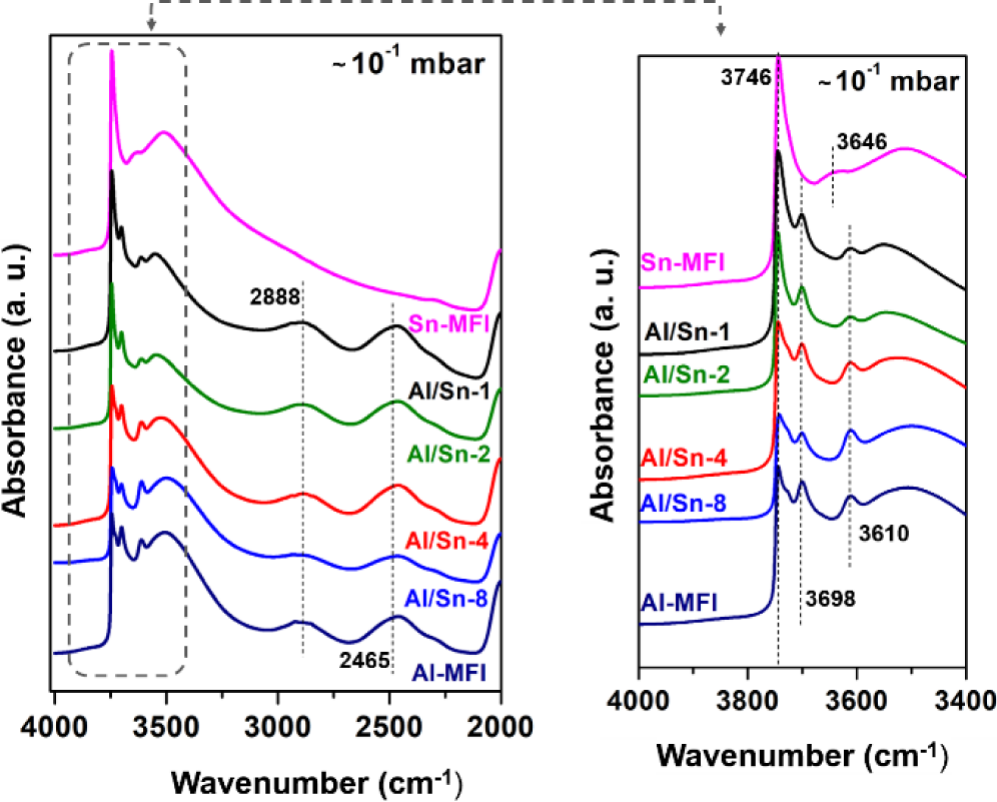
IR spectra after water adsorption on all the studied samples at ∼10^–1^ mbar.

As presented for Sn-MFI, the water adsorption for Al/Sn samples was also investigated by IR spectra at different pressures of water (Figure S15). A comparison between the spectra collected for all the samples is shown in Figure 3. The bands observed at 3746 and 3610 cm^–1^ correspond to silanol groups located at the external surface and the stretching mode of bridging OH groups (Si–OH-Al) in the zeolite, respectively. The intensity of the band at 3610 cm^–1^ drops continuously with increased water pressure, due to adsorption of water on the BAS_(Al)_ (Figure S16).

At the pressure of ∼10^–3^ mbar the additional bands at 3698, 2888, 2465, and 1630 cm^–1^ appear.

The band at 3698 cm^–1^ was attributed to unperturbed OH stretching vibrations, from H-bonded water adsorbed.^25^ In the difference spectra (Figure S16), the negative band observed at 3610 cm^–1^ for all samples with Al are related to the Bro̷nsted acid sites interacting with water, while the bands at 2888 cm^–1^, 2465 and 1630 cm^–1^ were corresponding to the A,B,C-triplet of the Fermi resonance, which is related with water molecules hydrogen bonded to BAS groups.^13^ The spectra of Sn-MFI, when compared with Sn/Al, exhibit a complete absence of the bands related to the Fermi resonance A,B,C-triplet. This absence suggests significant differences in the local chemical environment or coordination geometry of water at the Sn^4+^ sites in these materials.

### 2.4 Dehydration of Cyclohexanol in the Aqueous Phase

In aqueous phase, dehydration of alcohol is catalyzed by hydronium ions.^12^ For zeolite these hydronium ions are related to the BAS_(Al)_.^2,12,26−28^ The impact of the LAS sites on this reaction is unexplored. Table 2 summarizes the rate and turnover frequency (rate normalized to the concentration of BAS_(Al)_) observed at 150 °C and 40 bar (Figure S17).

**Figure 4:**
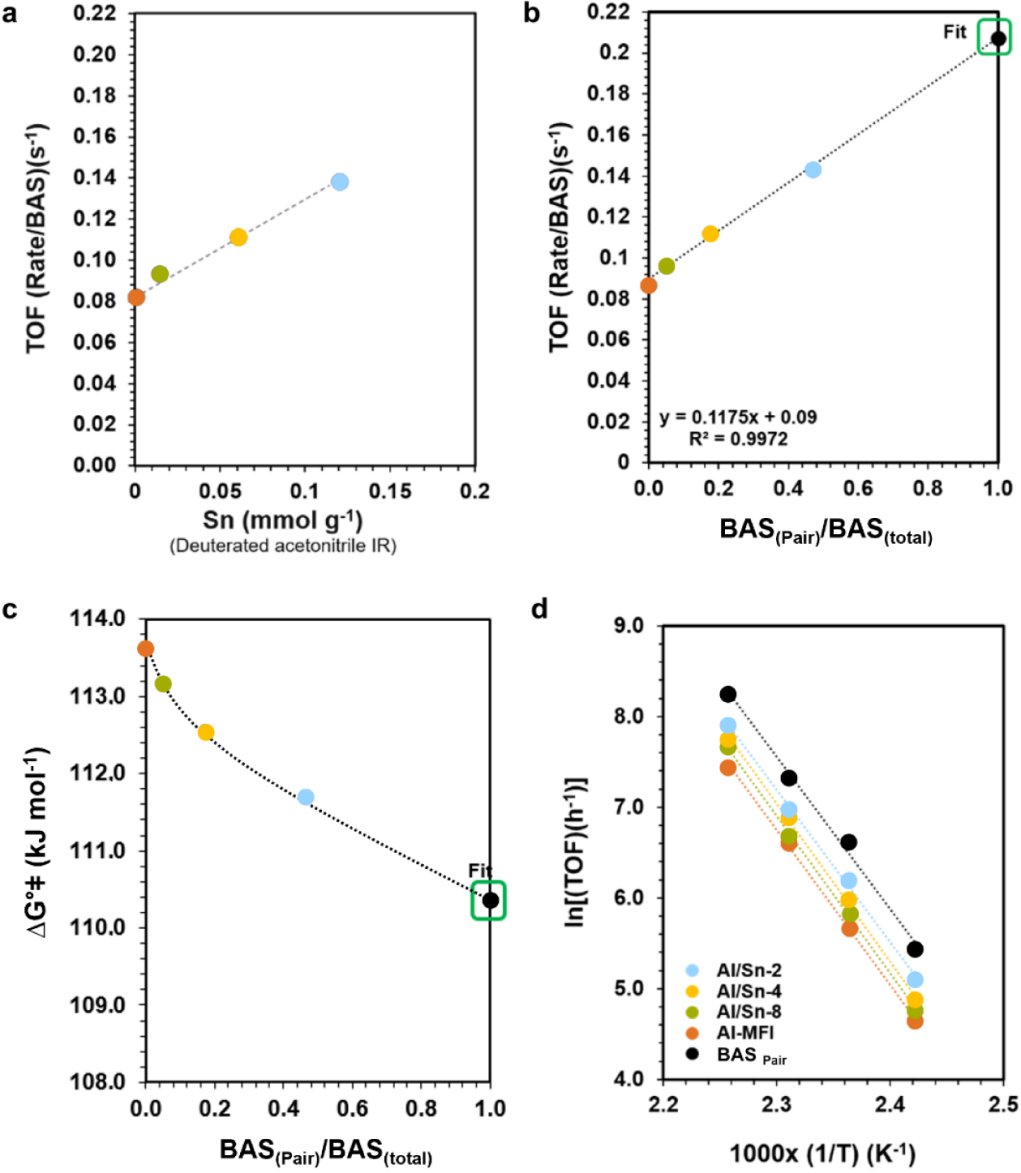
(a) TOF (rate/BAS_(Al)_) for cyclohexanol dehydration over Al/Sn-MFI samples as a function of the Sn concentration measured by CD_3_CN titration. Reaction conditions: at 150 °C, 40 bar of N_2_, 700 rpm, 0.33 M cyclohexanol–water solution. (b) TOF (rate/BAS_(Al)_) for cyclohexanol dehydration over Al/Sn-MFI samples as a function of BAS_(Pair)_/BAS_(total)_ (for detailed calculation, see SI). (c) Δ*G*°⧧ (Gibbs energy) at 150 °C as a function of the BAS_(Pair)_/BAS_(total)_. The average error for the Δ*G*°⧧ is ±0.2 kJ/mol (∼0.2%). (d) Arrhenius plot for the aqueous phase dehydration of cyclohexanol to cyclohexene over Al/Sn-MFI at 140 °C, 150 °C, 160, and 170 °C. Reaction conditions: cyclohexanol (3.3 g), H_2_O (100 mL), 150 °C, 40 bar H_2_, stirred at 700 rpm. (The dashed lines serve as a guide to the eye.).

Cyclohexene was the main product of cyclohexanol dehydration on all the zeolites. The reaction rate was independent of the concentration of cyclohexanol, i.e., showing a zero-order dependence (Figure S18). The aluminum-free sample (Sn-MFI) had very low catalytic activity, which is attributed to the absence of H_3_O^+^_hydr._ inside the pores of this zeolite. For Al/Sn samples, the rate increased with the concentration of Sn, also increasing the turnover frequency (TOF) (Figure 4) by a factor of 2. It should be noted in passing that the impact was smaller for Al/Sn-1 because of the lower crystallinity of Al/Sn-1. The TOF increased linearly with the fraction of BAS_(Pair)_ in the total BAS (Scheme S1; Figure 4b). Extrapolation of the trend allowed estimation of the specific TOF of these paired sites (Figures 4b, Figure S19).

The activation energies were similar for all zeolites with values between 140 and 150 kJ mol^–1^ (Figure S20 and Table 2), which indicates that the presence of Sn framework does not affect the BAS_(Al)_ acid strength, but slightly decreased Δ*G*°⧧ (Figure 4c).

The kinetic measurements indicated that all the samples may follow an E1 mechanism, as previously reported.^29^ In this mechanism, first, the adsorbed cyclohexanol is protonated, followed by water elimination, which involves the intermediate carbenium ion with stepwise C–O and C–H bond scissions. For the H-MFI studied samples, the protonation of the alcohol requires a hydronium ion, which only exists with the presence of BAS_(Al)_. As all the Sn/Al samples studied in this work have a similar concentration of BAS_(Al)_, the increase observed on the measured TOF values is concluded to be caused by LAS_(Sn)_. It should be emphasized that Sn sites in the absence of BAS_(Al)_ do not catalyze the reaction to an appreciable extent.

**Scheme 1:**
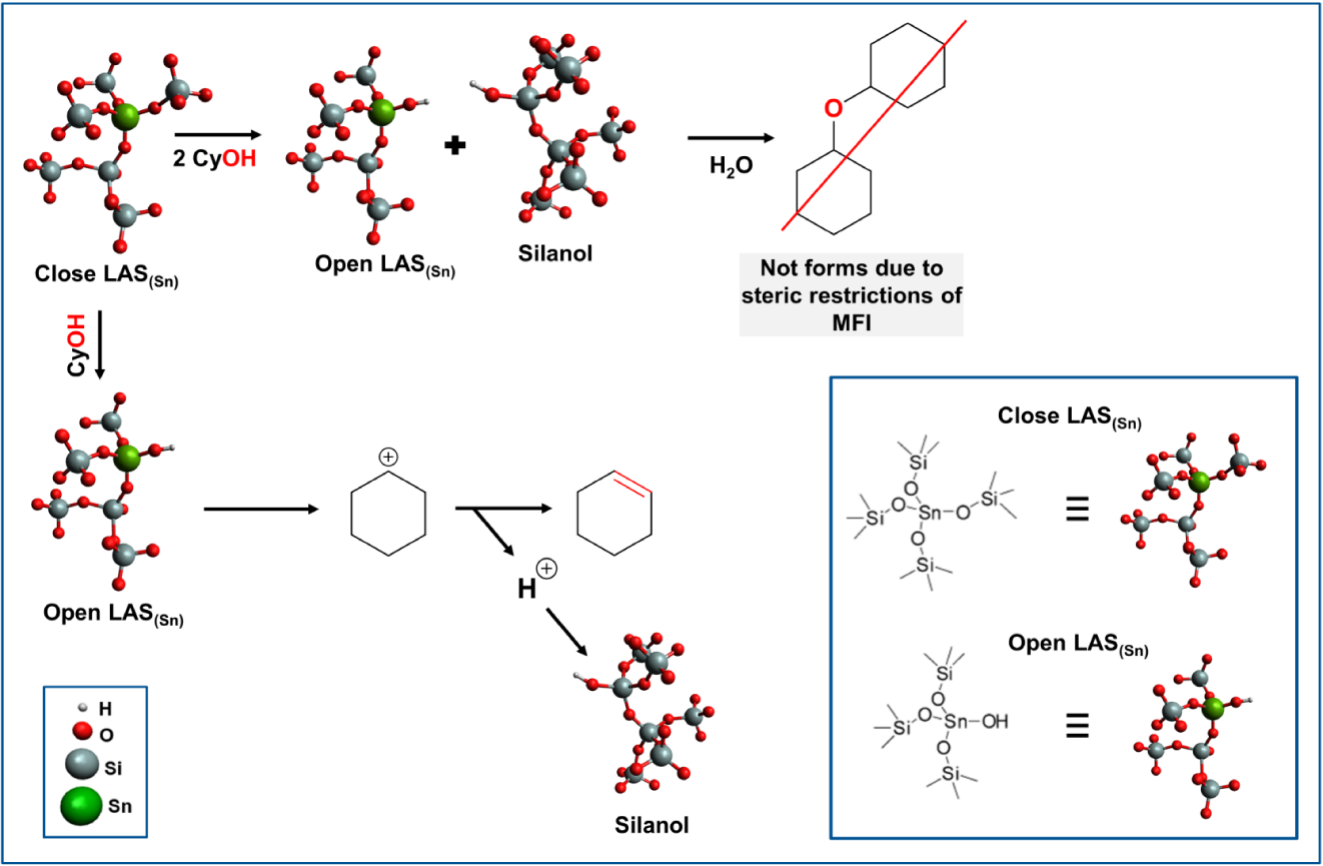
Hypothesized Mechanism of LAS_(Sn)_ for Dehydration of Cyclohexanol in Aqueous Phase

Conceptually, the carbenium ion intermediates could also undergo a bimolecular rearrangement and form an diphenyl ether molecule.^30^ The pore size of the MFI structure excludes this pathway (Scheme 1).

It is speculated that the abundance of water in aqueous phase leads to blocking of Sn^4+^, which may catalyze cyclohexanol dehydration in the absence of water.^31^ The higher rates in the presence of Sn^4+^ are, thus, caused by an increasing the pre-exponential factor (Figures 4d, Figure S20) in the presence of BAS_(Pair)_. This increase points to a later transition state in the C–O bond cleavage, which remains uncompensated by the back-donation of the proton to the neutral water cluster. Thus, the overall kinetic barrier is lower with respect to standard free energies, but remains unchanged in the standard enthalpy of the equilibrated intermediates plus the transition state.

To consider a possible confinement effect caused by the presence of LAS_(Sn)_, which may decrease the cyclohexanol space by increasing the concentration of water, we calculated the distance between two hydronium ions neighbors (*D*_h-h_) (see Scheme S2, Table S7 and Table S8). In general, the values were not strongly affected by the presence of the Sn. One also could argue that the presence of framework Sn may increase the Bro̷nsted acidity by an additional stabilization of the negative charge of zeolite framework or the polarization of an OH group.^32^ However, Shi et al.^12^ have shown that the dehydration of alcohol in the aqueous phase is independent of the intrinsic strength of the dissolved acid since the hydrated hydronium ions may be the most acidic species in aqueous acidic solutions.

**Figure 5:**
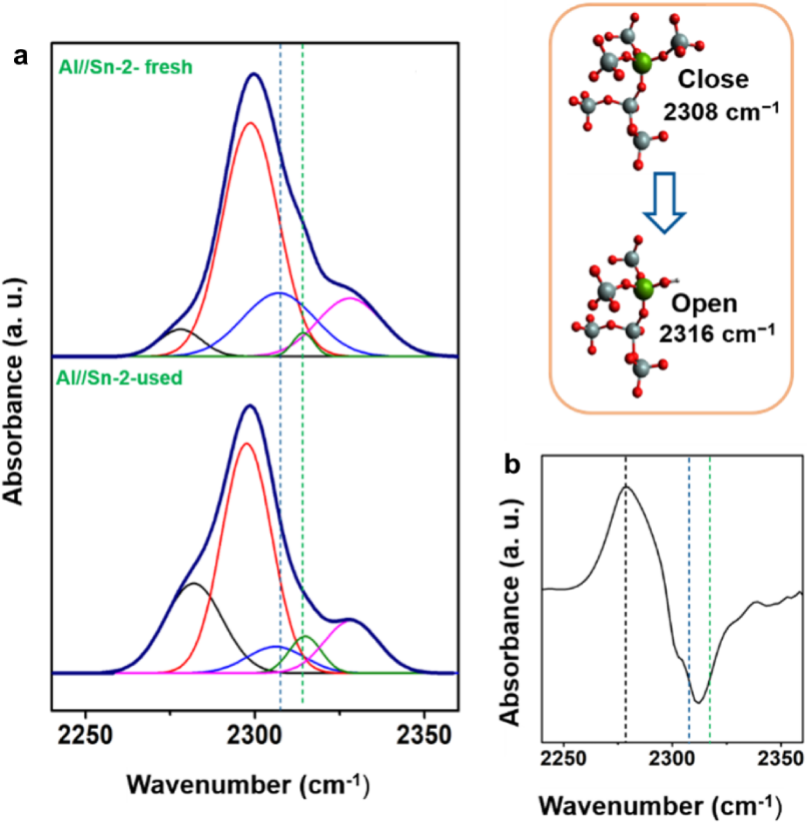
(a) IR spectra after CD_3_CN titration of the Al/Sn-2 fresh sample and the used one. The sample was applied in the reaction of dehydration of cyclohexanol at the liquid phase (150 °C, 20 min, 40 bar). Both samples were activated at 450 °C for 1 h followed by CD_3_CN titration at 40 °C. Si–OH/black, BAS_(Al)_/red, LAS_(EFAL)_/pink, open LAS_(Sn)_/blue, close LAS_(Sn)_/green. The deconvolution of the IR spectra was done with the ORIGIN software program using Gaussian-type curves. (b) Comparison of the difference between the IR spectra of the used samples after CD_3_CN titration and the spectrum of the fresh sample after CD_3_CN titration for Al/Sn-2.

Aiming to get a better visualization of the distribution of the acid sites in the reaction environment, IR spectra of used samples were collected and compared with the fresh ones (Figure S21). The peak at 3745 cm^–1^ is related to silanol groups, and the calculation of this area on the corresponding IR spectra indicates an increase in the Si–OH groups for the used Al/Sn-2, while it was almost constant for the Al-MFI sample. A similar tendency was observed after the titration with CD_3_CN of the Sn/Al-2 and Al/MFI used samples (Figure 5). The IR spectra of the used samples (Sn/Al-2 and Al-MFI) showed evidence of hydrolyzing the fully framework coordinated tin atoms (closed Sn) to framework tin centers (open Sn) after the reaction. For Sn/Al-2, the comparison between the fresh and used samples showed that after the reaction, the concentration of silanol groups (black dashed curve, 2275 cm^–1^) and the concentration of open framework Sn (green dashed curve, 2316 cm^–1^) increased, while the peak related to close framework Sn (blue dashed curve, 2308 cm^–1^) decreased.

The profiles observed for used Al-MFI were very similar to the ones collected for the fresh sample (Figure S22 and Table S9). The presence of these polar hydroxyl defect groups (Sn–OH) with BAS properties (BAS_(Sn)_), in close contact with BAS_(Al)_, may alter the thermodynamic state of the reagents inside the micropore environment. According to Choudhary et al.^33^ glucose molecules next to the metal center (LAS) that are bonded with OH (Bro̷nsted base) group will replace water molecules in the first shell of the metal center with its OH groups, by hydrogen donation. A similar mechanism has been identified for Sn-BEA-catalyzed aldose-to-ketose isomerization. It should be noted in passing that, this protonation indicates that oxygen around Sn^4+^ acts as Bro̷nsted base, deprotonating the alcohol hydroxyl group.^34^

The results demonstrate that the positive effect of Sn^4+^ on the rate of cyclohexanol dehydration is not caused by the direct catalytic action of Sn^4+^. It is also not caused by adjustment of the pore around the active site or an increase in the BAS acidity. The linear correlation between BAS_(Pair)_ and TOF values, combined with the slight, but decreasing tendency observed for Δ*G*°⧧ (Figure 4c) point to the role of Sn^4+^ that allows orientation of the polar group in cyclohexanol and a late transition state that is associated with a higher transition entropy. It should be noted in passing that such effects are also attributed to local fields induced by Lewis acid sites.

## 3 Conclusions

A series of hydrothermally synthesized MFI zeolites containing increasing concentrations of Sn^4+^ has been used to show the impact of Lewis acid sites in the proximity of Bro̷nsted acid sites. X-ray diffraction and the IR spectra of CD_3_CN showed all Sn to be located inside the zeolite lattice. The adsorption of water hydrolyzed the framework coordinated Sn atoms to the framework Sn centers with the formation of weak BAS_(Sn)_. However, without the presence of BAS_(Al)_, the samples were not able to catalyze the dehydration of cyclohexanol in the aqueous phase.

Although LAS_(Sn)_ did not catalyze the dehydration of cyclohexanol in the aqueous phase by itself, it enhanced the reaction rate at H_3_O^+^(H_2_O)_*n*_. This enhancement was caused by an increase in the transition entropy, which is currently attributed to a later transition state of the elimination caused by stabilization of the polar hydroxyl group and positively charged hexyl carbenium ion. This action of the cation mimics directional effects observed in enzyme pockets. It remains an open question whether this positive effect on the transition entropy could also be caused by local fields induced via Sn^4+^.

In summary, the observed impact of Sn^4+^ cations clearly indicates the potential and limitations of such modifications by incorporated heteroatoms for acid catalyzed reactions.

## 4 Experimental Section

### 4.1 Catalyst Preparation

A hydrothermal method was employed for the synthesis of Al/Sn-MFI zeolites.^21^ In a typical preparation, 15.3 g of tetraethyl orthosilicate (TEOS, Sigma-Aldrich, 99%) was hydrolyzed with 16.5 g of tetrapropylammonium hydroxide (TPAOH, Sigma-Aldrich, 99%, 40% solution in water) under vigorous stirring. Then, a solution of SnCl_4_ x H_2_O (Sigma-Aldrich 99%) and Al(NO_3_)_3_ x 9H_2_O (Sigma-Aldrich, 99%) was added to hydrolyze the TEOS. The molar composition of the final batch was Si/(1): Sn/(0.002–0.03): TPAOH/(0.45): Al/(0.02): H_2_O/(35) (Table S1). After stirring overnight, the clear gel was placed in an autoclave (300 mL) in an oven at 160 °C for 3 days. After crystallization, the material was filtered, washed with deionized water, dried at 100 °C and calcined in synthetic air (100 mL min^–1^) at 550 °C (1 °C min^–1^) for 5h. (The samples were named according to the molar Al/Sn ratio.)

### 4.2 Catalyst Characterization

Elemental analysis was performed on an Agilent 760 ICP-OES spectrometer. X-ray diffraction analysis was performed on a diffractometer, using Cu Kα radiation and operating at 45 kV and 40 mA. The scanning range was 2θ = 5°–50° with step increments of 0.01°. The relative crystallinity of the samples was calculated by comparing the total area of the two strong diffraction peaks in the 2θ region of 5°–35° with those from the Al-MFI sample (free of Sn). The unit cell parameters were calculated by using the UnitCell software. Surface areas and micropore volumes were determined by N_2_ adsorption at liquid N_2_ temperature with a Sorptomatic 1990 automated surface area and a pore size analyzer. Raman spectra were obtained using a Renishaw inVia Reflex Raman System. The laser line at 785 nm of a He–Cd laser was used as an exciting source with an output of 50 mW. The infrared spectra of the samples were recorded on a ThermoScientific Nicolet Fourier transform infrared (FT-IR) spectrometer. 120 Scans were accumulated for each spectrum. Before each IR experiment, the samples were activated in a vacuum (*p* = 10^–7^ mbar) at 450 °C for 1 h (10 °C min^–1^). After the mixture was cooled, a spectrum of the activated sample was collected.

The titration of the BAS and LAS experiments were performed using pyridine, acetonitrile-d3 (CD_3_CN), and water adsorption. After the activation (similar for all the analyses), the sample was equilibrated with vapor of pyridine (150 °C), CD_3_CN (40 °C), and water (35 °C) for 60 min followed by outgassing for 1 h, after which an IR spectrum was recorded (for more details please see Supporting Information). The water adsorption at the gas phase was performed using a Seteram microbalance connected to a vacuum system. In a typical experiment, the Al/Sn zeolite samples were loaded on the microbalance and activated under vacuum (<10^–4^ mbar) at 450 °C for 1 h and cooled to 30 °C afterward. The water vapor was introduced stepwise through a dosing valve. After equilibration under a certain pressure of water and at room temperature, the adsorbed amount was quantified by the mass increase, and the released heat was monitored by DSC signal. The total water uptake for all the samples inside the micropore was determined based on the point when the heat of adsorption approached ∼45 kJ mol^–1^, i.e., where the water started to condensate outside the pore.

### 4.3 Catalytic Reaction

The aqueous phase dehydration reactions were performed in a 300 mL Hastelloy Parr reactor. Initially, 100 mL of an aqueous cyclohexanol solution (0.33M) and a specific amount of catalyst (range 50–150 mg) were added to the reactor. In sequence, the reactor was pressurized with 40 bar H_2_ at room temperature and heated up to desired reaction temperature. The initial time of the reaction was based on the point at which the set temperature was reached. The agitation used was ∼700 rpm. At the end of the reaction, the reactor was cooled to room temperature. The reaction mixture of cyclohexene (oil phase) and cyclohexanol-containing aqueous phase was extracted with several fractions of CH_2_Cl_2_ (total amount was 100 mL). The solution was analyzed on an Agilent 7890A gas chromatograph (GC). For quantification, 1,3-dimethoxybenzene was used as an internal standard. The carbon balance was higher than 92%.

## Supporting Information Available

Syntheses of the MFI studied, characterizations, and reactional analysis details (Scheme S1–S2, Table S1–S9, Figure S1–S22) (PDF)

## References

[ref1] Gounder R.; Davis M. E. Beyond Shape Selective Catalysis with Zeolites: Hydrophobic Void Spaces in Zeolites Enable Catalysis in Liquid Water. AichE J. 2012, 59, 3349–3358. 10.1002/aic.14016.

[ref2] Eckstein S.; Hintermeier P. H.; Zhao R.; Baráth E.; Shi H.; Liu Y.; Lercher J. A. Influence of Hydronium Ions in Zeolites on Sorption. Angew. Chem., Int. Ed. 2019, 58, 3450–3455. 10.1002/anie.201812184. 30600885

[ref3] Shamzhy M.; Opanasenko M.; Concepción P.; Martínez A. New Trends in Tailoring Active Sites in Zeolite-Based Catalysts. Chem. Soc. Rev. 2019, 48, 1095–1149. 10.1039/C8CS00887F. 30624450

[ref4] Taramasso M.; Milanese S. D.; Perego G.; Milan B. N.. Preparation of porous crystalline synthetic material comprised of silicon and titanium oxides. US Patent, US 4,410,501 A, 1983, 1 8.

[ref5] Boronat M.; Concepción P.; Corma A.; Renz M.; Valencia S. Determination of the Catalytically Active Oxidation Lewis Acid Sites in Sn-Beta Zeolites and their Optimisation by the Combination of Theoretical and Experimental Studies. J. Catal. 2005, 234, 111–118. 10.1016/j.jcat.2005.05.023.

[ref6] Kornatowski J.; Wichterlová B.; Jirkovský J.; Löffler E.; Pilz W. Spectroscopic Studies of Vanadium-Substituted Zeolitic Silicates of MFI Topology. J. Chem. Soc., Faraday Trans. 1996, 92 (6), 1067–1078. 10.1039/FT9969201067.

[ref7] Yang G.; Zhou L.; Han X. Lewis and Brönsted Acidic Sites in M^4+^-Doped Zeolites (M = Ti, Zr, Ge, Sn, Pb) as well as Interactions with Probe Molecules: A DFT Study. J. Mol. Catal. A: Chem. 2012, 363–364, 371–379. 10.1016/j.molcata.2012.07.013.

[ref8] Harris J. W.; Cordon M. J.; Di Iorio J. R.; Vega-Vila J. C.; Ribeiro F. H.; Gounder R. Titration and Quantification of Open and Closed Lewis Acid Sites in Sn-Beta Zeolites that Catalyze Glucose Isomerization. J. Catal. 2016, 335, 141–154. 10.1016/j.jcat.2015.12.024.

[ref9] Shahami M.; Ransom R.; Shantz D. F. Synthesis and Characterization of Tin, Tin/Aluminum, and Tin/Boron Containing MFI Zeolites. Microporous Mesoporous Mater. 2017, 251, 165–172. 10.1016/j.micromeso.2017.05.049.

[ref10] Courtney T. D.; Chang C. C.; Gorte R. J.; Lobo R. F.; Fan W.; Nikolakis V. Effect of Water Treatment on Sn-BEA Zeolite: Origin of 960 cm^–1^ FTIR Peak. Microporous Mesoporous Mater. 2015, 210, 69–76. 10.1016/j.micromeso.2015.02.012.

[ref11] Mei D.; Lercher J. A. Effects of Local Water Concentrations on Cyclohexanol Dehydration in H-BEA Zeolites. J. Phys. Chem. C 2019, 123, 25255–25266. 10.1021/acs.jpcc.9b07738.

[ref12] Shi H.; Eckstein S.; Vjunov A.; Camaioni D. M.; Lercher J. A. Tailoring Nanoscopic Confines to Maximize Catalytic Activity of Hydronium Ions. Nat. Commun. 2017, 8, 1–7. 10.1038/ncomms15442. 28541290 PMC5458516

[ref13] Vjunov A.; Wang M.; Govind N.; Huthwelker T.; Shi H.; Mei D.; Fulton J. L.; Lercher J. A. Tracking the Chemical Transformations at the Bro̷nsted Acid Site upon Water-Induced Deprotonation in a Zeolite Pore. Chem. Mater. 2017, 29, 9030–9042. 10.1021/acs.chemmater.7b02133.

[ref14] Yang X.; Wang F.; Wei R.; Li S.; Wu Y.; Shen P.; Wang H.; Gao L.; Xiao G. Synergy Effect between Hierarchical Structured and Sn-Modified H[Sn, Al]ZSM-5 Zeolites on the Catalysts for Glycerol Aromatization. Microporous Mesoporous Mater. 2018, 257, 154–161. 10.1016/j.micromeso.2017.08.039.

[ref15] Xia C.; Liu Y.; Lin M.; Peng X.; Zhu B.; Shu X. Confirmation of the Isomorphous Substitution by Sn Atoms in the Framework Positions of MFI-Typed Zeolite. Catal. Today 2018, 316, 193–198. 10.1016/j.cattod.2018.02.056.

[ref16] Sun W.; Wang X.; Yang J.; Lu J.; Han H.; Zhang Y.; Wang J. Pervaporation Separation of Acetic Acid-Water Mixtures through Sn-Substituted ZSM-5 Zeolite Membranes. J. Membr. Sci. 2009, 335, 83–88. 10.1016/j.memsci.2009.02.037.

[ref17] Trombetta M.; Armaroli T.; Alejandre A. G.; Solis J. R.; Busca G. An FT-IR Study of the Internal and External Surfaces of HZSM5 Zeolite. Appl. Catal. A Gen. 2000, 192, 125–136. 10.1016/S0926-860X(99)00338-5.

[ref18] Kustov L. M.; Kazanskii V. B.; Beran S.; Kubelková L.; Jiru P. Adsorption of carbon monoxide on ZSM-5 zeolites: infrared spectroscopic study and quantum-chemical calculations. J. Phys. Chem. 1987, 91, 5247–5251. 10.1021/j100304a023.

[ref19] Mendes P. G.; Moreira M. L.; Tebcherani S. M.; Orlandi M. O.; Andrés J.; Li M. S.; Diaz-Mora N.; Varela J. A.; Longo E. SnO2 Nanocrystals Synthesized by Microwave-Assisted Hydrothermal Method: Towards a Relationship between Structural and Optical Properties. J. Nanoparticle Res. 2012, 14, 750 10.1007/s11051-012-0750-7.

[ref20] Pelmenschikov A. G.; Van Santen R. A.; Jänchen J.; Meijer E. CD3CN as a Probe of Lewis and Bronsted Acidity of Zeolites. J. Phys. Chem. 1993, 97, 11071–11074. 10.1021/j100144a028.

[ref21] Yuan E.; Dai W.; Wu G.; Guan N.; Hunger M.; Li L. Facile Synthesis of Sn-Containing MFI Zeolites as Versatile Solid Acid Catalysts. Microporous Mesoporous Mater. 2018, 270, 265–273. 10.1016/j.micromeso.2018.05.032.

[ref22] Wichterlová B.; Tvarůžková Z.; Sobalík Z.; Sarv P. Determination and Properties of Acid Sites in H-Ferrierite: A Comparison of Ferrierite and MFI Structures. Microporous Mesoporous Mater. 1998, 24, 223–233. 10.1016/S1387-1811(98)00167-X.

[ref23] Roy S.; Bakhmutsky K.; Mahmoud E.; Lobo R. F.; Gorte R. J. Probing Lewis Acid Sites in Sn-Beta Zeolite. ACS Catal. 2013, 3, 573–580. 10.1021/cs300599z.

[ref24] Josephson T. R.; Jenness G. R.; Vlachos D. G.; Caratzoulas S. Distribution of Open Sites in Sn-Beta Zeolite. Microporous Mesoporous Mater. 2017, 245, 45–50. 10.1016/j.micromeso.2017.02.065.

[ref25] Jentys A.; Warecka G.; Derewinski M.; Lercher J. A. Adsorption of Water on ZSM5 Zeolites. J. Phys. Chem. 1989, 93, 4837–4843. 10.1021/j100349a032.

[ref26] Foo G. S.; Wei D.; Sholl D. S.; Sievers C. Role of Lewis and Bro̷nsted Acid Sites in the Dehydration of Glycerol over Niobia. ACS Catal. 2014, 4, 3180–3192. 10.1021/cs5006376.

[ref27] Wang Z.; Wang L.; Jiang Y.; Hunger M.; Huang J. Cooperativity of Bro̷nsted and Lewis Acid Sites on Zeolite for Glycerol Dehydration. ACS Catal. 2014, 4, 1144–1147. 10.1021/cs401225k.

[ref28] Zhao C.; He J.; Lemonidou A. A.; Li X.; Lercher J. A. Aqueous-Phase Hydrodeoxygenation Of Bio-Derived Phenols To Cycloalkanes. J. Catal. 2011, 280, 8–16. 10.1016/j.jcat.2011.02.001.

[ref29] Liu Y.; Vjunov A.; Shi H.; Eckstein S.; Camaioni D. M.; Mei D.; Baráth E.; Lercher J. A. Enhancing the Catalytic Activity of Hydronium Ions through Constrained Environments. Nat. Commun. 2017, 8, 2–9. 10.1038/ncomms14113. 28252021 PMC5337972

[ref30] Bukowski B. C.; Bates J. S.; Gounder R.; Greeley J. First Principles, Microkinetic, and Experimental Analysis of Lewis Acid Site Speciation during Ethanol Dehydration on Sn-Beta Zeolites. J. Catal. 2018, 365, 261–276. 10.1016/j.jcat.2018.07.012.

[ref31] Corma A.; Domine M. E.; Valencia S. Water-Resistant Solid Lewis Acid Catalysts: Meerwein-Ponndorf-Verley and Oppenauer Reactions Catalyzed by Tin-Beta Zeolite. J. Catal. 2003, 215, 294–304. 10.1016/S0021-9517(03)00014-9.

[ref32] Li G.; Pidko E. A. The Nature and Catalytic Function of Cation Sites in Zeolites: A Computational Perspective. ChemCatchem 2019, 11, 134–156. 10.1002/cctc.201801493.

[ref33] Choudhary V.; Pinar A. B.; Lobo R. F.; Vlachos D. G.; Sandler S. I. Comparison of Homogeneous and Heterogeneous Catalysts for Glucose-to-Fructose Isomerization in Aqueous Media. ChemSuschem 2013, 6, 2369–2376. 10.1002/cssc.201300328. 24106178

[ref34] Li G.; Pidko E. A.; Hensen E. J. M. Synergy between Lewis Acid Sites and Hydroxyl Groups for the Isomerization of Glucose to Fructose over Sn-Containing Zeolites: A Theoretical Perspective. Catal. Sci. Technol. 2014, 4, 2241–2250. 10.1039/C4CY00186A.

