# Correction to “Impact of Sn Lewis Acid Sites on the Dehydration of Cyclohexanol”

**DOI:** 10.1021/acscatal.4c05977

**Published:** 2024-10-28

**Authors:** Karen A. Resende, Ruixue Zhao, Yue Liu, Eszter Baráth, Johannes A. Lercher

Page 11747: The acknowledgment should read as given here.

