# Supplementary material for: Impact of Sn Lewis Acid Sites on the Dehydration of Cyclohexanol
Source: ACS Catal. 2024 Jul 24;14(15):11741–8. doi: 10.1021/acscatal.4c01608 (PMC11301620; doi:10.1021/acscatal.4c01608)
Supplement: Supplementary file 1 — cs4c01608_si_001.pdf [file cs4c01608_si_001.pdf]

Supporting Information

Impact of Sn Lewis Acid Sites on the Dehydration of Cyclohexanol

Karen A. Resende,[a] Ruixue Zhao,[a] Yue Liu,[a] Eszter Baráth,[a,b,*] Johannes A. Lercher[a,c,*]

[a]Department of Chemistry and Catalysis Research Center, Technische Universität München, Lichtenbergstrasse 4, 85748 Garching, Germany

[b]Current address: Leibniz-Institut für Katalyse e. V. (LIKAT Rostock), Albert-Einstein-Str. 29a, D-18059 Rostock, Germany

[c]Institute for Integrated Catalysis, Pacific Northwest National Laboratory, Richland, WA 99354, USA

*E-mail: eszter.barath@catalysis.de; johannes.lercher@ch.tum.de

Content

1. Experimental S3
1.1. Catalyst preparation S3
1.2. Catalyst characterization S3
BET S3
Titration of acid sites S3
IR- Pyridine followed by water adsorption S3
Cyclohexanol adsorption S3
2. Results and Discussion S4
2.1. Structural characterization S4
2.2. Quantification of acid sites S7
2.3. Hydrophobic / Hydrophilic domains- Water adsorption S8
2.3.1. Water adsorption: Linear combination S13
2.3.2. BAS-Sn ($BAS_{(Pair)}$) calculations S14
2.4. Dehydration of cyclohexanol in the aqueous phase S21
2.4.1. Calculation of the distance between two hydronium ions neighbors S23
3. References S25

1. Experimental

1.1. Catalyst preparation

Table S1. Molar ratio used for the syntheses of the studied MFI samples; Si/Sn and Si/Al ratios; Compositions. Si/Al=50 was the same for all the samples.

Samples	$SnCl_4$	$Al(NO_3)_3$	TPAOH	TEOS	H_2O	Si/Sn	Sn* (mmol g^{-1})	Al** (mmol g^{-1})	Al/Sn[a]
Sn-MFI	0.03	-	0.45	1	35	33	0.51	0.00	-
Al/Sn-0.7	0.03	0.02	0.45	1	35	33.3	0.4	0.35	0.7
Al/Sn-1	0.02	0.02	0.45	1	35	50	0.35	0.35	1
Al/Sn-2	0.01	0.02	0.45	1	35	100	0.17	0.35	2
Al/Sn-4	0.005	0.02	0.45	1	35	200	0.08	0.35	4
Al/Sn-8	0.0025	0.02	0.45	1	35	400	0.04	0.35	8
Al-MFI	0	0.02	0.45	1	35	--	0.00	0.35	-

*Values were calculated based on the synthesis procedure.
**Values were measured by elemental analysis.
[a]Molar ratio.

1.2. Catalyst characterization

BET. Before the measurements, all samples were evacuated at a pressure of 10–2 mbar and treated at 250 °C for 2 h. The total surface area was calculated by the Brunauer-Emmett-Teller (BET) equation, while the micropore size distribution was determined using the t-plot method. For these calculations, the error of the measurement is < 1%.

Titration of acid sites. For the titration with pyridine, the concentrations of Brønsted and Lewis acid sites were calculated from the integral intensities of the peaks at 1540 and 1450 cm^{-1}, respectively.[1] The integrated molar extinction coefficient (E) for this IR peak were: 1.42 (LAS) and 1.67 (BAS).[2] For the titration with CD_3CN the peaks overlapped so the deconvolution of the peak in the wavenumber range 2360-2240 cm^{-1} was performed. The following peaks were considered: Sn Lewis acid sites inside the framework: open (2316 cm^{-1}) and closed (2308 cm^{-1}),[3] hydrogen-bound to Si-OH sites (2275 cm^{-1}), BAS (2297 cm^{-1})[3] and Al-LAS (2225 cm^{-1}).[3] The integrated molar extinction coefficient (E) for these IR peaks was used according to Harris et al.[2] The number of Lewis acid sites (total) and BAS were coherent between pyridine and CD_3CN titanium IR experiments. For the titration with water, the pressure inside the cell was raised from 10^{-3} to 1 mbar, after equilibration the spectra were collected.[4]

IR- Pyridine followed by water adsorption. Initially, Sn-MFI was activated in a vacuum (p = 10^{-7} mbar) at 450 °C for 1 h (10 °C min^{-1}). After cooling the cell, a spectrum of the activated sample was collected. The titration of the BAS and LAS experiments were performed using pyridine adsorption at 150 °C. In sequence, the sample was cooled down to 30°C under 10^{-6} mbar vacuum. Then the water adsorption was performed at different pressures (from 10^{-5} to 10^{-1}). In sequence, the sample was heated to 150 °C and titrated again with pyridine. For the titration with pyridine, the concentrations of Brønsted and Lewis acid sites were calculated from the integral intensities of the peaks at 1540 and 1450 cm^{-1}, respectively.

Cyclohexanol adsorption. The adsorption of cyclohexanol from aqueous phase were performed for Al/Sn-2 sample. In a typical experiment 300 mg of zeolite were immersed in 5 mL of cyclohexanol aqueous solution and kept under agitation at least 24 hours at 30 °C. The cyclohexanol concentration were measured before and after the adsorption on an Agilent 7890A gas chromatograph (GC). The adsorption measurements were carried out in a Setaram Calvet C80 calorimeter.[5]

2. Results and Discussion

2.1. Structural characterization

(A)

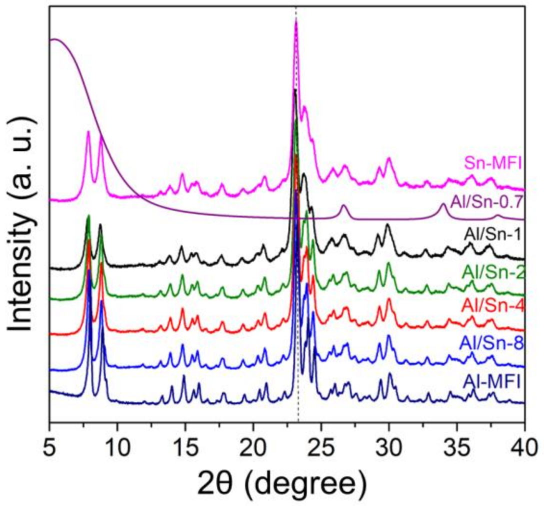

(B)

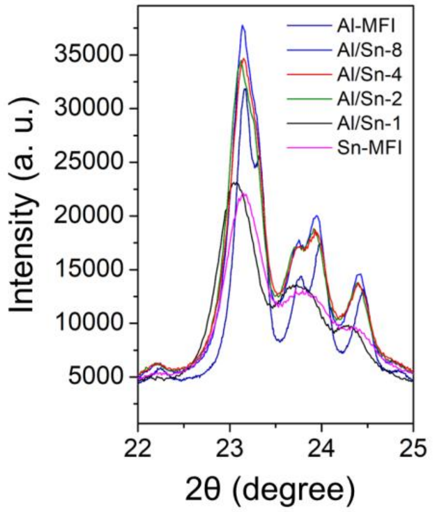

Figure S1. (A) Powder XRD patterns of the Sn/Al samples prepared in this study in 2θ = 5°-40° range. The XRD profiles were normalized. **(B)** Comparison of the non-normalized XRD profiles of the studied samples among the XRD profiles for 2θ = 22°-25° range.

Table S2. Calculated unit cell volume used crystal system and atomic lattices of each synthesized zeolite. The calculation unit cell parameters were performed using the UnitCell software, which is a least-squares refinement program to retrieve unit cell constants from diffraction data. The followed plans are considered: (101), (011), (200), (020), (102), (301), (501), (431), (303), (033), (352) and (541). Parameter value sigma 95%.

Sample	a (Å)	b (Å)	c (Å)	Crystal system	Volume $(Å)^3$
*MFI $_{ICSD-280364}$	20.05109	19.87569	13.36823	Orthorhombic	5327.63
Al-MFI	20.06671	19.94768	13.40490	Orthorhombic	5365.7744
Al/Sn-8	20.04908	19.94454	13.42024	Orthorhombic	5366.3466
Al/Sn-4	20.05318	19.95549	13.41991	Orthorhombic	5370.2610
Al/Sn-2	20.07641	19.99399	13.42954	Orthorhombic	5390.7185
Al/Sn-1	20.10458	20.02208	13.50732	Orthorhombic	5449.3634
Sn-MFI	20.04323	19.95982	13.43503	Orthorhombic	5403.7305

*MFI samples without heteroatoms.

Table S3. Physicochemical properties of Sn/Al-MFI measured by N_2 adsorption-desorption: BET-surface area; Micropore volume and mesopore area.

Samples	BET surface area (m^2 g^{-1})	Mesopores surface area (m^2 g^{-1})
Sn-MFI	381	110
Al/Sn-1	450	139
Al/Sn-2	440	126
Al/Sn-4	448	84
Al/Sn-8	406	90
Al-MFI	353	62

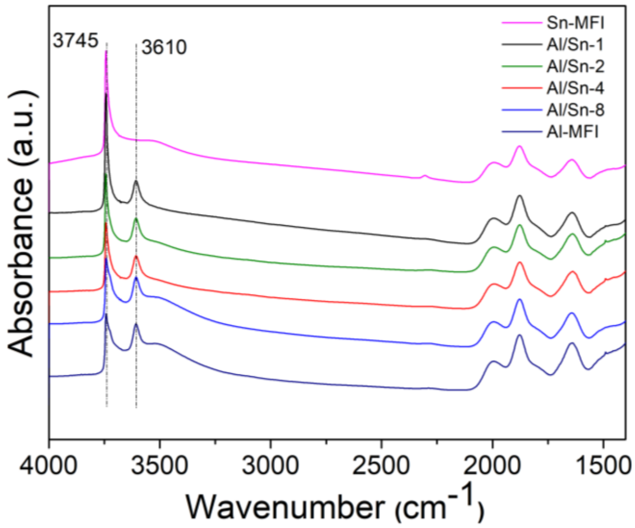

Figure S2. IR spectra of the Al/Sn substituted MFI samples after activation at 450 °C for 1h. The spectra were collected at 150°C under vacuum (10^{-6} mbar).

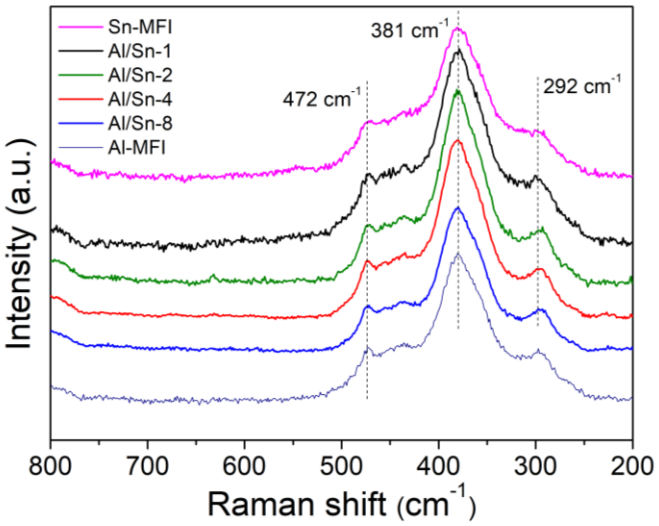

Figure S3. Characterization of calcined Sn/Al MFI zeolite samples.

2.2. Quantification of acid sites

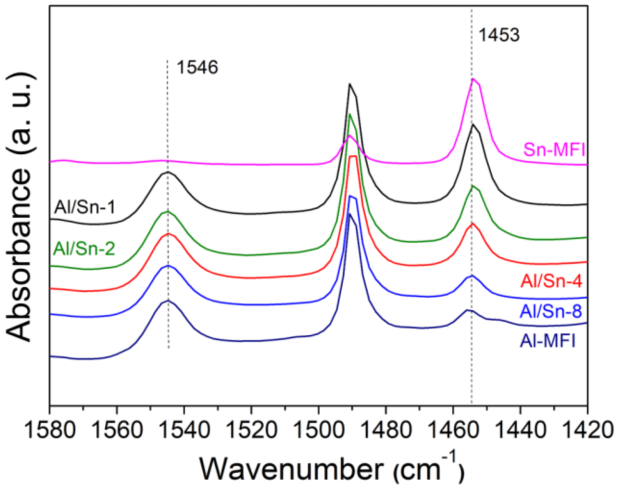

Figure S4. IR spectra after titration of pyridine at 150 °C on the Sn/Al-MFI zeolites prepared in this study. All the samples were activated at 450 °C for 1h.

2.3. Hydrophobic / Hydrophilic domains - Water adsorption

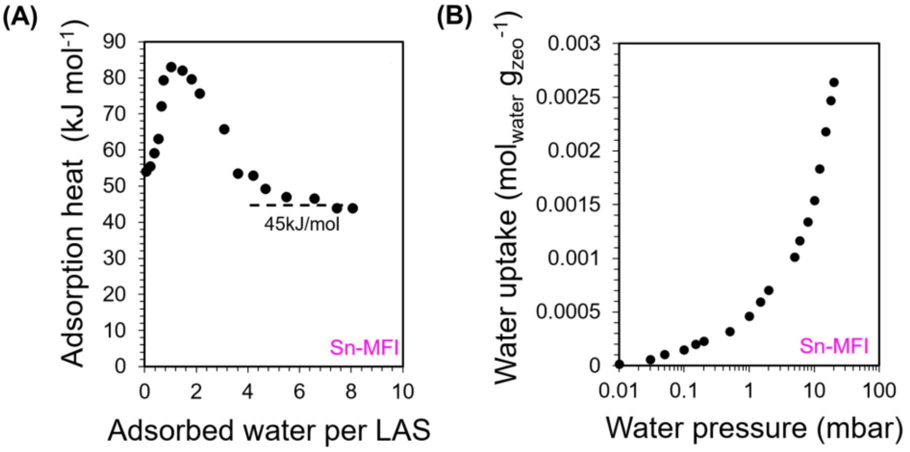

Figure S5. (A) Adsorption heat of water on Sn-MFI zeolites as a function of water per LAS. **(B)** Adsorption isotherm of water from gas phase on Sn-MFI zeolite at room temperature.

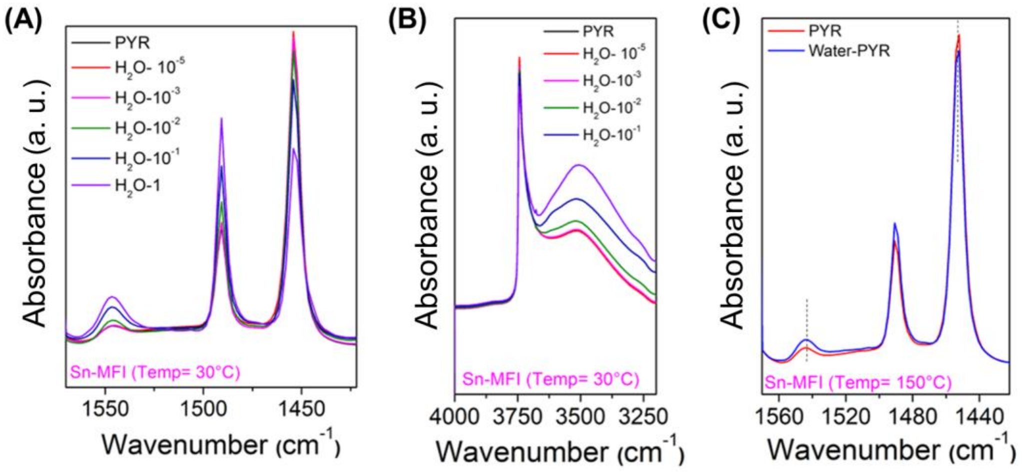

PYR-IR-150°C<br>Acid sites (μmol g^{-1})		
**Sample**	**BAS**	**LAS**
Sn-MFI	9.8	330
Sn-MFI *water	29.3	308

Figure S6. (A) IR spectra of water adsorption for Sn-MFI at pressure range: 10^{-5}- 1 mbar^{-1} and 30°C in the followed wavenumber range: 1565-1430 cm^{-1}. **(B)** IR spectra of water adsorption for Sn-MFI at pressure range: 10^{-5}- 1 mbar^{-1} and 30 °C in the followed wavenumber range: 4000-3000 cm^{-1}. **(C)** Comparison between the IR spectra after titration of pyridine at 150 °C and IR spectra after water followed by another titration of pyridine at 150 °C for Sn-MFI.

(A)

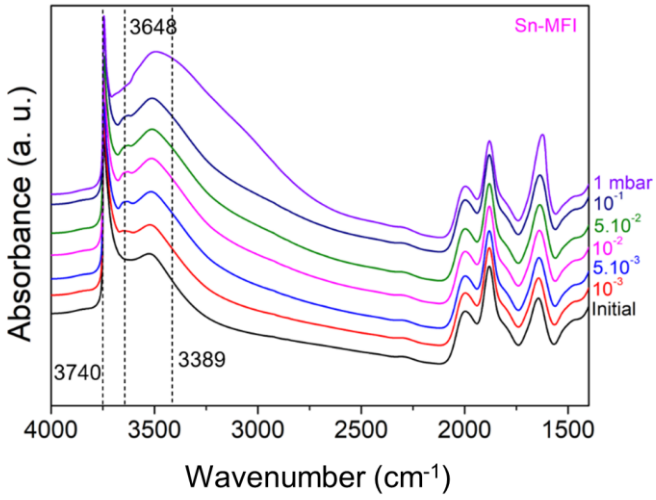

(B)

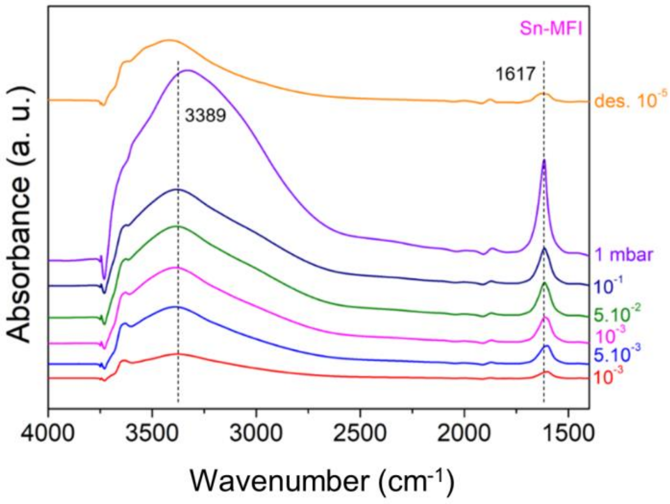

Figure S7. (A) IR spectra of water during on Sn-MFI sample at 10^{-3}-1mbar pressure range. **(B)** Difference between the IR spectra of the adsorbate with water at the equilibrium pressure (indicated) and the spectrum Sn-MFI sample (fresh) after activation.

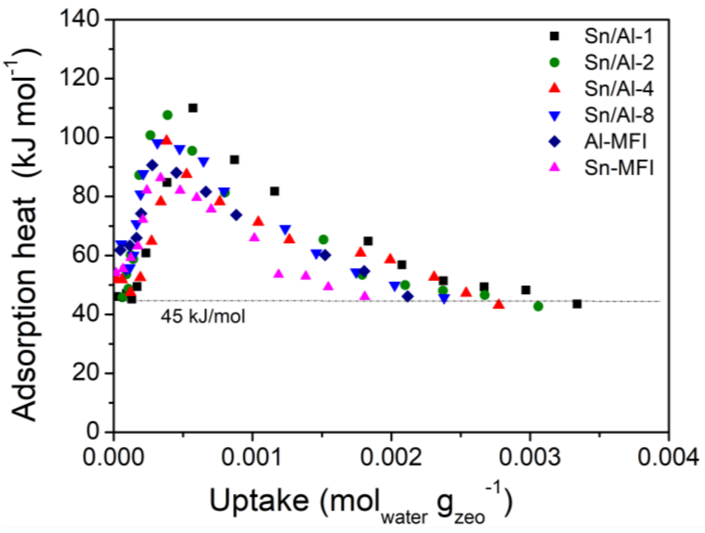

Figure S8. Adsorption heat of the water on the prepared zeolites (zeo) as a function of water uptake at room temperature.

Table S4. Water uptake from water gas phase adsorption on Sn/Al-MFI zeolites at room temperature.

**Sample**	**Total uptake***	**Water uptake BAS$_{(Al)}$***	**Water uptake LAS$_{(Sn)}$***	**Total micropore volume***	**Total micropore volume free water***	**Polar/Nonpolar**
Al-MFI	3.124	3.124	0	16.9	13.78	0.23
Al/Sn-8	4.078	3.124	0.95	18.0	13.93	0.29
Al/Sn-4	4.789	3.124	1.66	18.8	14.02	0.34
Al/Sn-2	5.197	3.124	2.07	16.3	11.10	0.45
Al/Sn-1	5.799	3.124	2.67	12.9	7.11	0.82

*Values multiplied by 10^{-2} and with a unit of cm^3 g^{-1}.

Water uptake. The residual water uptake for framework Sn (water uptake LAS$_{(Sn)}$) was calculated after accounting for binding water on BAS$_{(Al)}$ ~8 water molecule, according to equation 1 (eq 1), all values are in cm^3 g^{-1}. The total water uptake was collected at the point where the heat of vapor water adsorption achieved the heat of condensation water (~45 kJ mol^{-1}) (Figure S8).

$$\text{Water uptake LAS}_{(\text{Sn})} = \text{Total water uptake} - \text{Water uptake/BAS}_{(\text{Al})} \qquad \textbf{(eq 1)}$$

The total micropore volume free of water (cm^3 g^{-1}) was calculated according to equation 2.

$$\text{Volume free} = \text{Total micropore volume} - \text{Water uptake LAS}_{(\text{Sn})} - \text{Water uptake/BAS}_{(\text{Al})} \qquad \textbf{(eq 2)}$$

Total micropore volume was measured by adsorption of N_2 using the t-plot method. The polar and nonpolar domains were calculated as follows: Polar = Total water uptake and Nonpolar = Total micropore volume free of water.

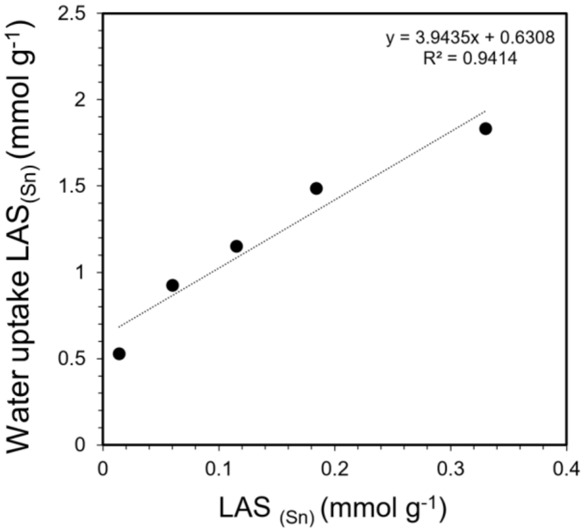

Figure S9. Molar vapor water uptake on $LAS_{(Sn)}$ at room temperature plotted as a function of the concentration of $LAS_{(Sn)}$ (mmol g^{-1}) inside the framework measured by titration with CD_3CN.

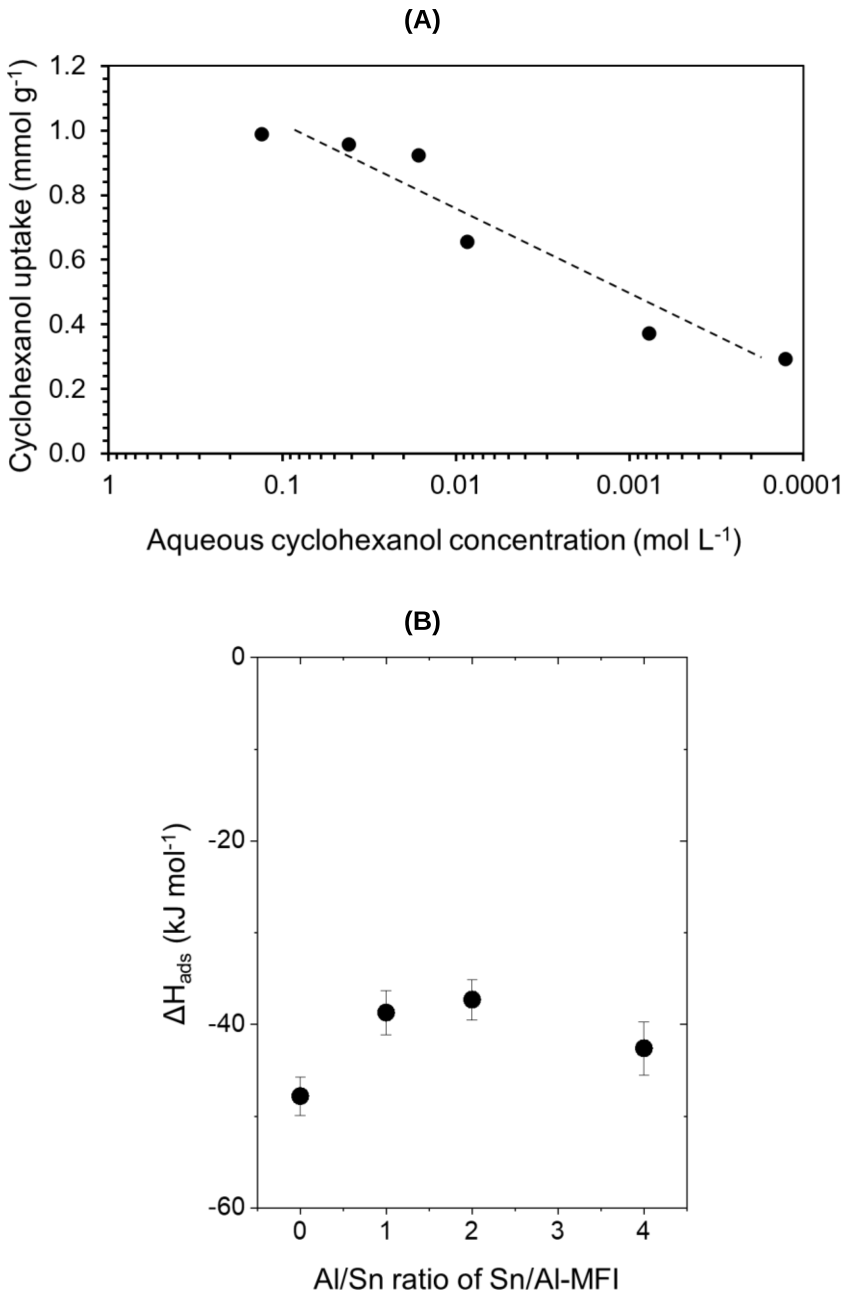

Figure S10. (A) CyOH uptake from aqueous phase adsorption on Al/Sn-2 at room temperature. **(B)** CyOH adsorption experiments: 5mM CyOH in water at room temperature.

Table S5. Adsorption properties of cyclohexanol on Al/Sn-2 at room temperature.

Sample	Sat. uptake (mmol g^{-1})	Sat. uptake* (cm^3 g^{-1})	Residual volume* (cm^3 g^{-1})	Adsorption heat (kJ mol^{-1})
**Al/Sn-2**	0.989	10.4	5.8	~40

***Values multiplied by 10^{-2} and with a unit of cm^3 g^{-1}.

Note: The presence of nearby Sn and Al for BAS pair formation was challenged to be characterized in the presence of water. Water adsorption experimental data clearly showed that zeolites substituted with different concentrations of Sn (and similar Al concentrations) had a significant increase in the adsorption of water capacity and strength in comparison to Al-MFI

(Table S4). This observation suggests a strong correlation between the concentration of BAS and the presence of Sn^{4+} ions in the zeolite structure.

2.3.1. Water adsorption: Linear combination

Linear combination of vapor water uptake. The hypothetical water uptake for each pressure was calculated according to equation 3.

$$\text{Water uptakeHyP [Pressure]} = \text{C\#Sn} * \left(\frac{\text{Water uptake(Sn)}}{\text{C(Sn)}}\right) + \text{C\#Al} * \left(\frac{\text{Water uptake(Al)}}{\text{C(Al)}}\right) \quad \textbf{(eq 3)}$$

C#Sn: Specific Sn concentration on the studied sample ($g_{Sn}\ g_{zeolite}^{-1}$);

C#Al: Specific Al concentration on the studied sample ($g_{Al}\ g_{zeolite}^{-1}$);

Water uptake (Sn): Water uptake for Sn-MFI (mol water $g_{zeolite}^{-1}$);

C(Sn): Concentration of Sn on Sn-MFI ($g_{Sn}\ g_{zeolite}^{-1}$);

Water uptake (Al): Water uptake for Al-MFI (mol water $g_{zeolite}^{-1}$);

C(Al): Concentration of Al on Al-MFI ($g_{Al}\ g_{zeolite}^{-1}$);

Linear combination of the vapor water adsorption heat. The hypothetical derivative heat was for each pressure calculated according to equation 4.

$$\text{Derivative heatHyP [Pressure]} = \frac{\text{C\#Sn}\left(\frac{\text{Integral heat(Sn)}}{\text{C(Sn)}}\right) + \text{C\#Al}\left(\frac{\text{Integral heat(Al)}}{\text{C(Al)}}\right)}{\text{(Mol water)HyP [Pressure]}} \quad \textbf{(eq 4)}$$

Integral heat (Sn): Integral heat for Sn-MFI (kJ).

Integral heat (Al): Integral heat for Al-MFI (kJ).

The hypothetical mol of water adsorbed for Sn and Al were calculated according to equation 3 and substituted at equation 5.

$$\text{(Mol water)HyP [Pressure]} = \text{C\#Sn}\left(\frac{\text{Mol water(Sn)}}{\text{C(Sn)}}\right) + \text{C\#Al}\left(\frac{\text{Mol water (Al)}}{\text{C(Al)}}\right) \quad \textbf{(eq 5)}$$

Mol water (Sn): Mols of water adsorbed at specific pressure for Sn-MFI.

Mol water (Al): Mols of water adsorbed at specific pressure for Al-MFI.

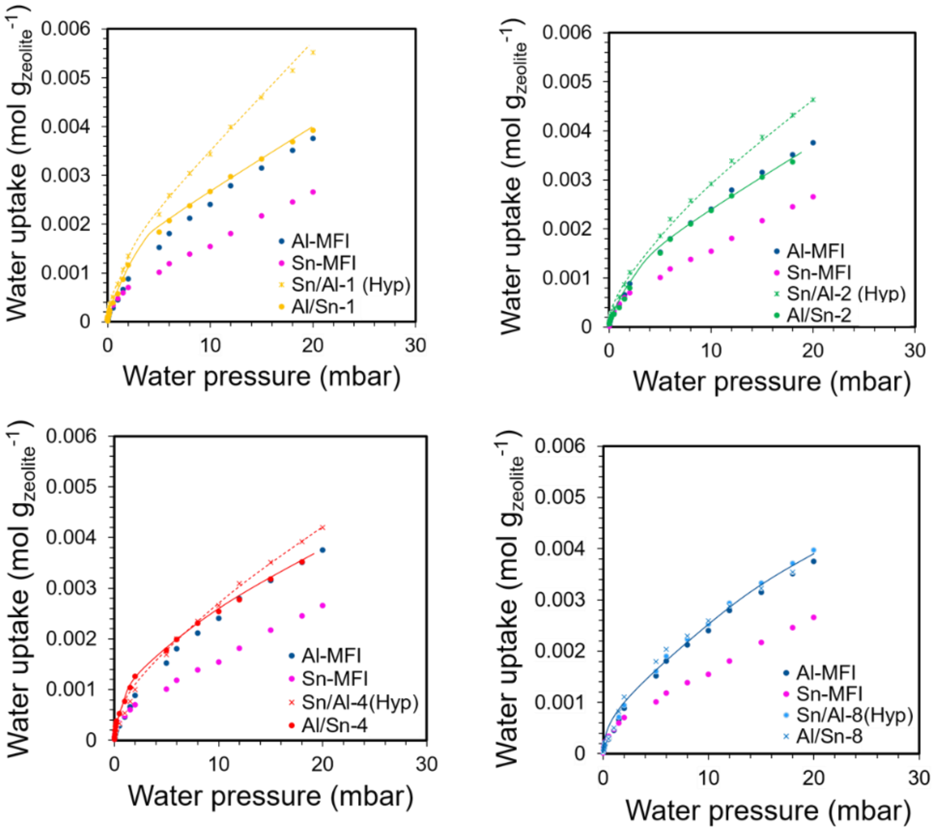

Figure S11. Adsorption isotherm of water from gas phase on Al/Sn-MFI zeolites samples, Al-MFI and Sn-MFI at room temperature and the respective linear combination.

2.3.2. BAS-Sn ($BAS_{(Pair)}$) calculations

The water uptake data from vapor water adsorption showed a clear interaction between the $BAS_{(Al)}$ and Sn framework. For a specific sample the $BAS_{(total)}$ will be a combination of normal $BAS_{(Al)}$ and BAS in close contact with framework Sn ($BAS_{(Pair)}$), as presented in Scheme S1 and equation 6. As known, $BAS_{(Al)}$ are stronger acid sites than with Sn sites framework isolated and silanol, so, hypothetically, at lower pressure the water will adsorb first on the Brønsted sites. Also, at low-pressure conditions, water may form a monolayer covered on $BAS_{(Al)}$, which was confirmed by the IR analysis during water adsorption.

$$[BAS_{(total)}] = [BAS_{(Al)}] + [BAS_{(Pair)}] \quad \textbf{(eq 6)}$$

Assuming the presence of normal $BAS_{(Al)}$ and BAS in close contact with framework Sn ($BAS_{(Pair)}$), the total water uptake as function of the pressure (P) (at lower pressure) was modeling as a combination of the water uptake on each of the BAS sites (equation 7).

$$[\text{Water uptake on } BAS_{(total)}](P) = [\text{Water uptake on } BAS_{(Al)}](P) + [\text{Water uptake on } BAS_{(Pair)}](P) \quad \textbf{(eq 7)}$$

At low-pressure (water uptake/$BAS_{(total)}$<1), with monolayer water covered and considering independent sites, the Langmuir equation was applied to represent the water uptake on each BAS (equation 8 and equation 9).

$$\frac{[\text{Water uptake on } BAS_{(Al)}](\text{P})}{BAS_{(Al)}} = Q_{(\text{Al})} = \frac{Qo_{(Al)}*Kl_{(Al)}*P}{1+ Kl_{(Al)}*P} \qquad \textbf{(eq 8)}$$

$$\frac{[\text{Water uptake on } BAS_{(Pair)}](\text{P})}{BAS_{(Pair)}} = Q_{(Pair)} = \frac{Qo_{(Pair)}*Kl_{(Pair)}*P}{1+ Kl_{(Pair)}*P} \qquad \textbf{(eq 9)}$$

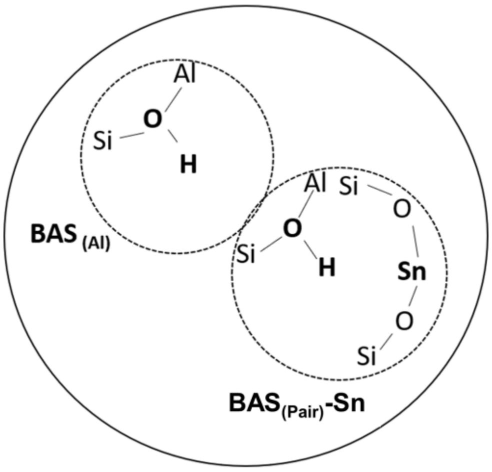

Scheme S1. Representation of $BAS_{(total)}$.

Where, $Kl_{(Al)}$ and $Kl_{(Pair)}$ are the Langmuir isotherm constants, which measures the water affinity to the adsorption sites; $Qo_{(Al)}$ and $Qo_{(Pair)}$ are the saturated monolayer coverage capacity for each BAS. In sequence, the water uptake on $BAS_{(Al)}$ and $BAS_{(Pair)}$ were isolated from the eq 8 and eq 9, respectively, and substituted in the eq 7, which resulted in eq10.

$$[\text{Water uptake on } BAS_{(total)}](P) = Qo_{(Al)}* BAS_{(Al)} + Qo_{(Pair)} * BAS_{(Pair)} \qquad \textbf{(eq 10)}$$

Next, using equation 6, $BAS_{(Al)}$ was substituted in equation 10, and all the parts were divided by BAS_{total}:

$$\frac{[\text{Water uptake on } BAS_{(total)}](\text{P})}{BAS_{(total)}} = \left[\frac{Qo_{(Al)}*Kl_{(Al)}*P}{(1+ Kl_{(Al)}*P)} * \frac{(1-BAS_{(Pair)})}{BAS_{(total)}}\right] + \left[\frac{Qo_{(Pair)}*Kl_{(Pair)}*P}{(1+ Kl_{(Pair)}*P)} * \frac{BAS_{(Pair)}}{BAS_{(total)}}\right] \qquad \textbf{(eq 11)}$$

In eq 11, the water uptake on $BAS_{(total)}$ for each sample was measured using the vapor water adsorption (data only at lower pressures were fitted.) The concentration of $BAS_{(total)}$ were measured by pyridine adsorption. To calculate the values for $Kl_{(Al)}$ and $Qo_{(Al)}$, the vapor water uptake for Al-MFI was used, since only $BAS_{(Al)}$ is presented in this sample. The water uptake as function of the pressure was normalized by the $BAS_{(total)}$ and applied to eq 8 (after linearization) (Figure S12).

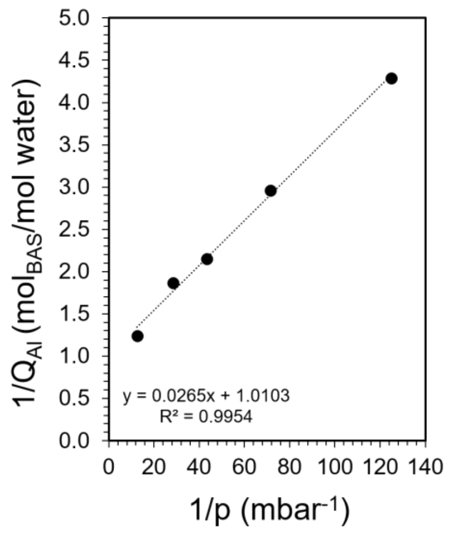

Figure S12. $1/Q_{Al}$ as function of 1/p. Parameters obtained from the linearization of the Langmuir equation for Al-MFI sample (equation 8).

The following constant values were obtained from this fit: $Kl_{(Al)}= 4.2 * 10^{-4}$ bar^{-1} and $Qo_{(Al)}= 0.989 \pm 0.09$ mol water mol BAS^{-1}.

In sequence, $Kl_{(Al)}$ and $Qo_{(Al)}$ were substituted in equation 11 and the vapor water uptake normalized by the $BAS_{(total)}$ were fitted to this equation for Al/Sn samples. $Qo_{(Pair)}$ and $Kl_{(Pair)}$ were considered to be equal for all Al/Sn samples during the fit. Table S6 shows the obtained values and Figure S15 the curves.

Table S6. Values obtained from the fit of the vapor water uptake normalized by the $BAS_{(total)}$ for each specific sample into the Equation 11.

**Samples**	**Residual^2**	**Regressed $BAS_{(Pair)}$/ $BAS_{(total)}$**	**$Kl_{(Pair)}$ $*10^{-4}$ (bar^{-1})**	**$Qo_{(Pair)}$**	**Sn/ $BAS_{(total)}$[a]**
Al/Sn-1	0.0009	0.210	1.3	3.133	0.88
Al/Sn-2	0.0009	0.469	1.3	3.133	0.55
Al/Sn-4	0.0006	0.175	1.3	3.133	0.29
Al/Sn-8	0.0014	0.049	1.3	3.133	0.07

[a] Real concentration of Sn divided by the concentration of $BAS_{(total)}$.

In general, there was a good correlation between the measured values and the fitted ones. We also presented the water vapor uptake curves related with $BAS_{(Al)}$ and $BAS_{(Pair)}$ separately.

Based on the $Qo_{(Pair)}$ obtained for the Al/Sn samples, we hypothesize that for $BAS_{(Pair)}$, initially one water may adsorb on each site, BAS and Sn. Next, as hydrated Sn sites may have two water molecules in the first coordination sphere,[6] it was hypothesized that the next water will adsorbed on Sn. This scenario will lead to a saturated monolayer coverage capacity for each $BAS_{(Pair)}$ equal to 3 water. In sequence, the next water my adsorbed on the BAS, which will lead to the protonation of this water and consequently the hydronium ion formation. This protonation is an exothermic process, that may contribute to the hydrolysis of the open Sn to close one,[7] as represented by Figure S13.

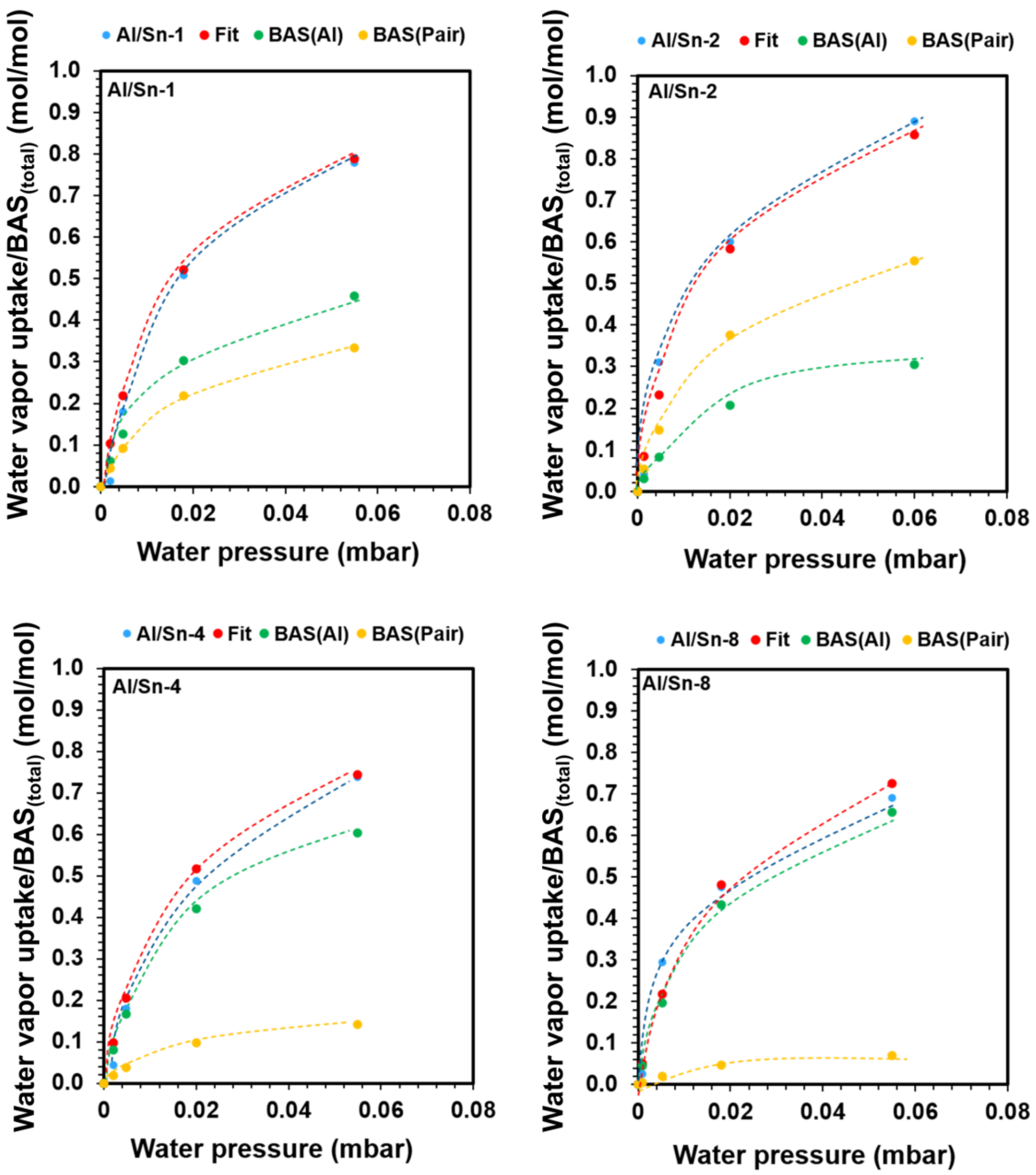

Figure S13. Curves obtained from the fit of the vapor water uptake normalized by the $BAS_{(total)}$ into the equation 11 for each specific sample. Blue points are the collected data, red are the fit values, green is the participation of the $BAS_{(Al)}$ and yellow are the participation of the $BAS_{(Pair)}$.

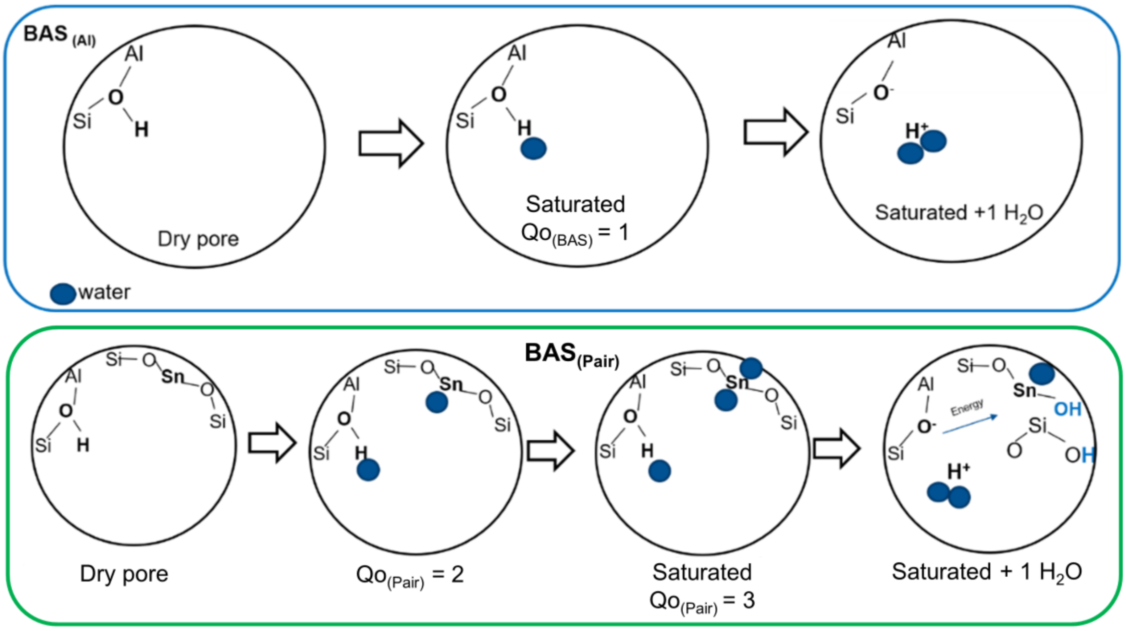

Figure S14. Scheme of the water adsorption on BAS(Al) and BAS(Pair) based on the fitted model.

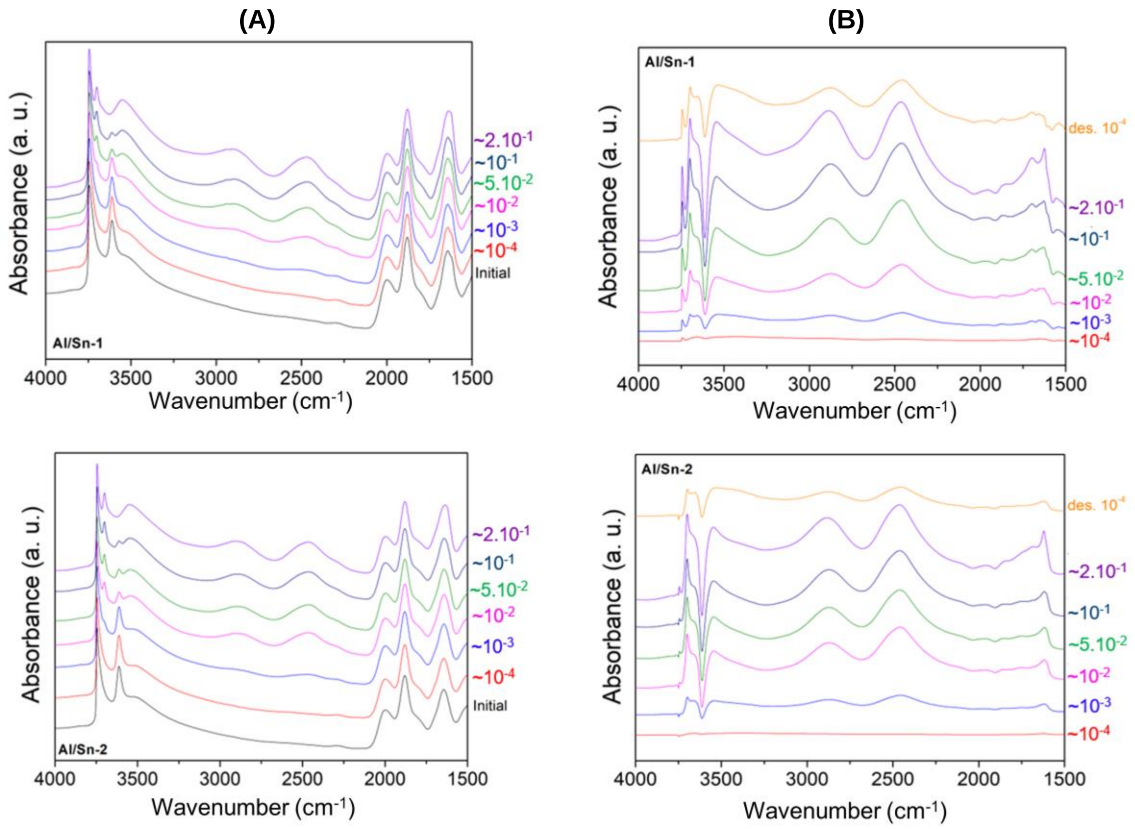

This figure continues on the next page (page S19).

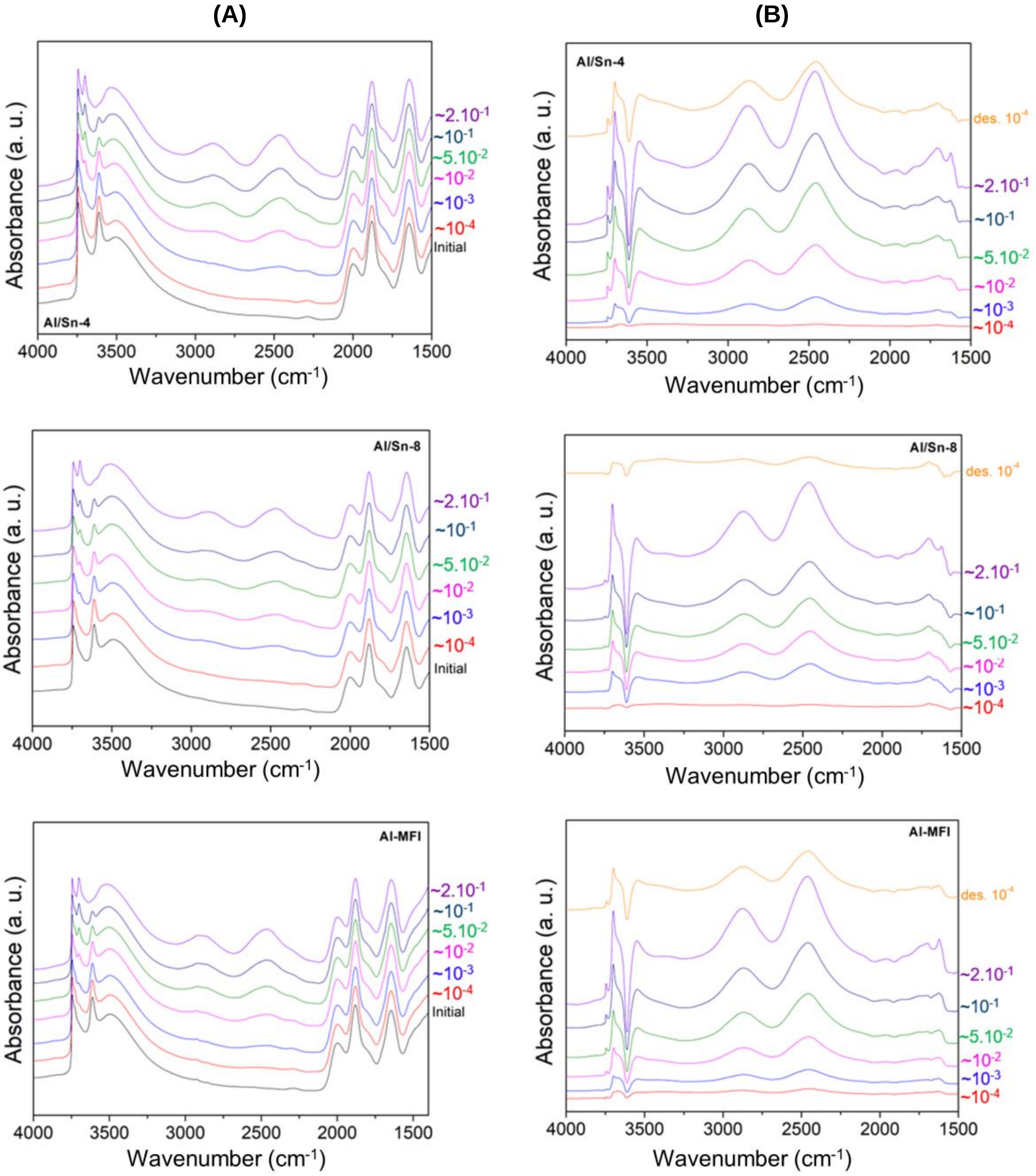

Figure S15. (A) IR spectra of water during on the MFI samples at 10^{-4}-10^{-1} mbar pressure range. **(B)** Difference between the IR spectra of the adsorbate with water at the equilibrium pressure (indicated) and the spectrum of the MFI samples (fresh) after activation.

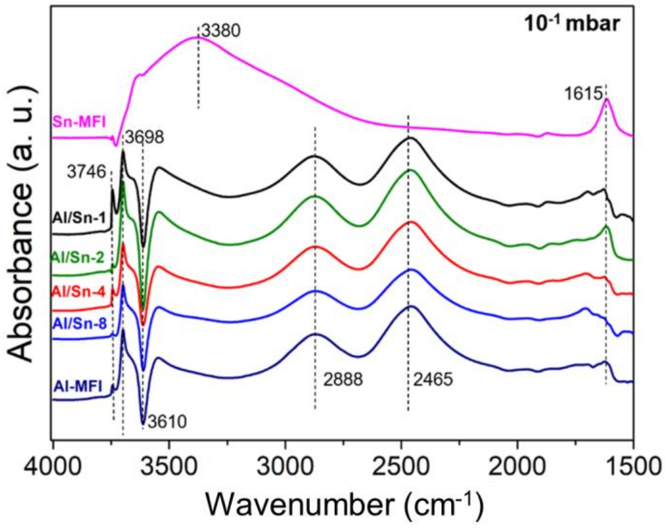

Figure S16. Comparison of the difference between the IR spectra after water adsorption at 10^{-1} mbar and the spectrum of the respective sample after activation.

2.4. Dehydration of cyclohexanol in the aqueous phase

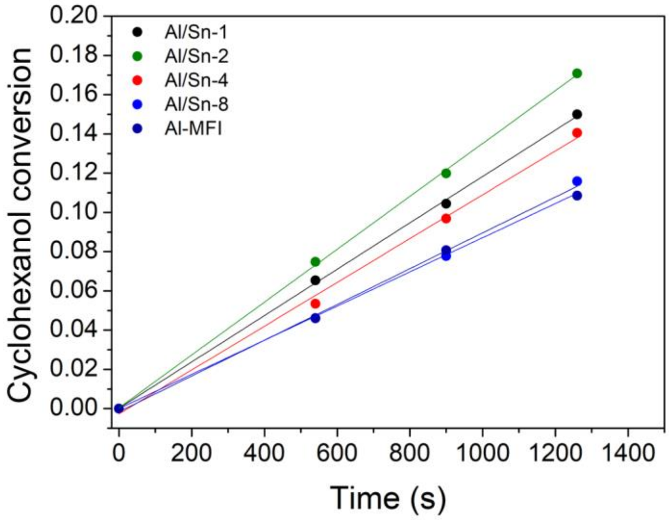

Figure S17. Aqueous phase dehydration of cyclohexanol to cyclohexene as a function of time. Reaction conditions: cyclohexanol (3.3 g), H_2O (100 mL), 150 °C, 40 bar H_2, stirred at 700 rpm.

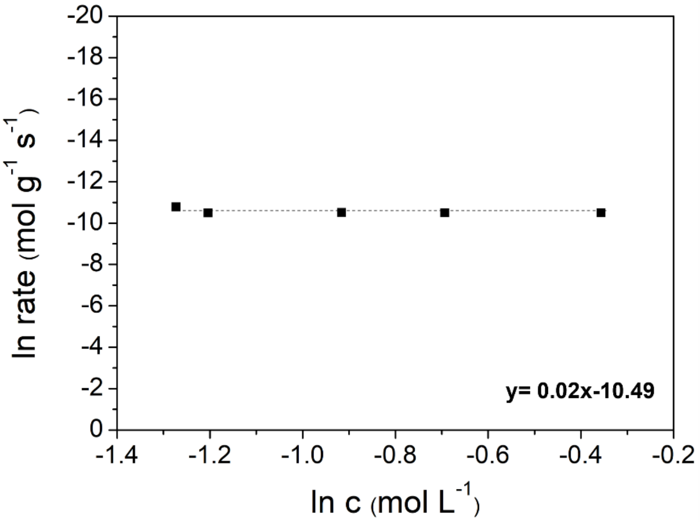

Figure S18. The dependence of the mass-specific reaction rate on the concentration of cyclohexanol for dehydration over Sn/Al-2 in the aqueous phase. Reaction conditions: cyclohexanol (2.8 g, 3.3 g, 4.4 g, 8.0 g, 10.0 g and 12.5 g), Sn/Al-2 (150 mg), water (100 mL), stirred at 700 rpm, 150°C. Aqueous concentrations of cyclohexanol higher than 7.7 M are not possible at 150 °C temperature due to the solubility of cyclohexanol in water.

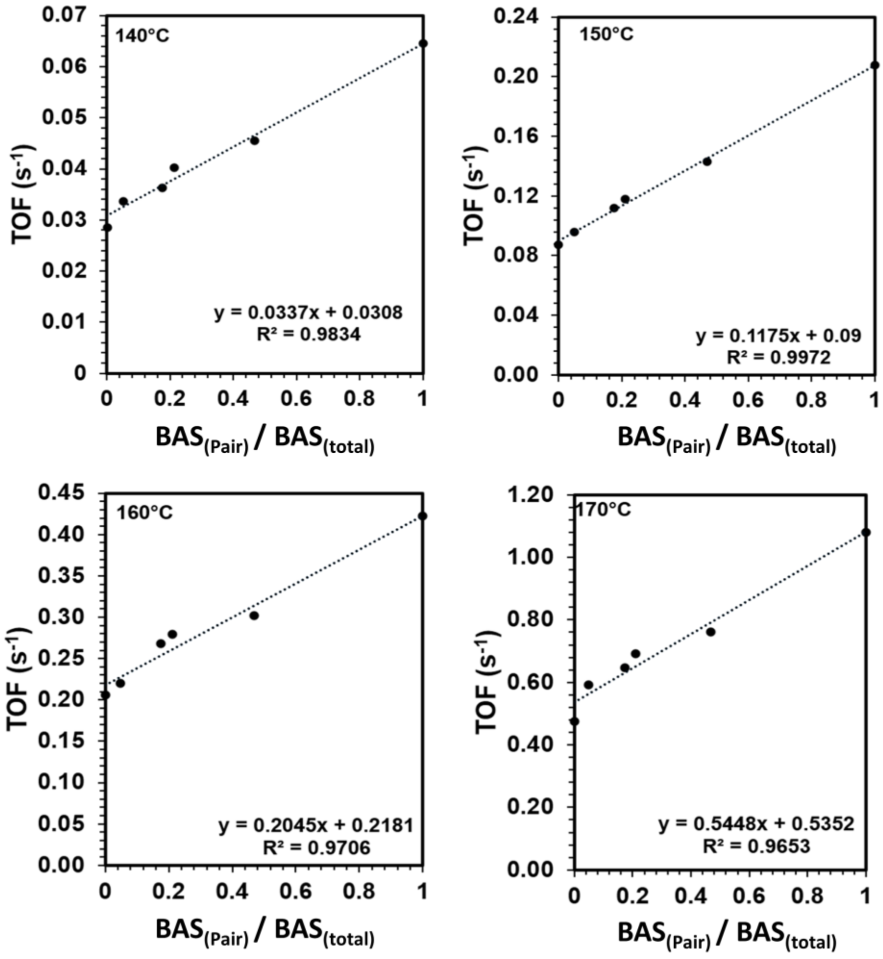

Figure S19. Reaction TOF (s^{-1}) versus the $BAS_{(Pair)}$/ $BAS_{(total)}$ at different temperature: 140-170 °C. The values of TOF at $BAS_{(Pair)}$/ $BAS_{(total)}$ = 1 was obtained using the fitted linear equation presented in each graphic.

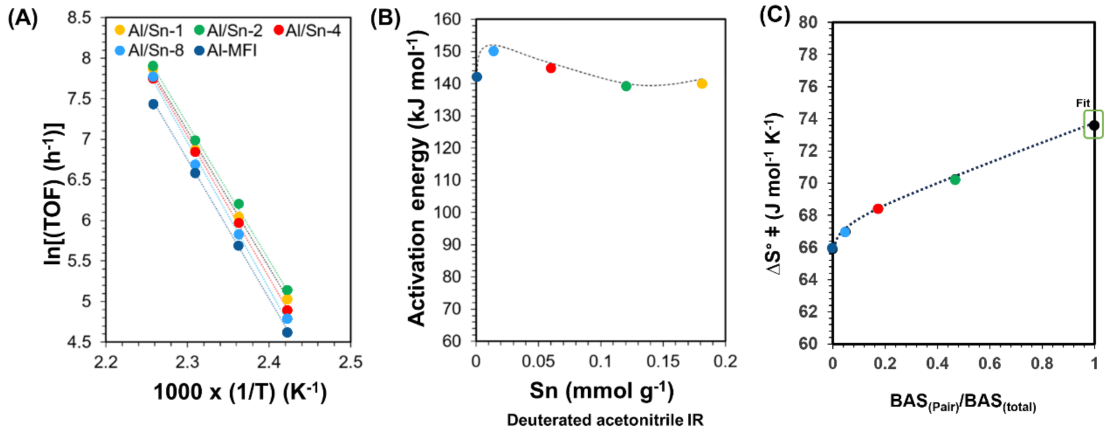

Figure S20. (A) Activation energy (Ea) calculation for the aqueous-phase dehydration of cyclohexanol to cyclohexene over Al/Sn MFI at 140 °C, 150 °C, 160 °C and 170 °C. Reaction conditions: Reaction conditions: cyclohexanol (3.3 g), H_2O (100 mL), 150 °C, 40 bar H_2, stirred at 700 rpm. **(B)** The activation energy (kJ mol^{-1}) as a function of the concentration of Sn inside the MFI framework studied determined by CN_3CD titration. **(C)** Entropy values calculated for the aqueous phase dehydration of cyclohexanol to cyclohexene over Al/Sn-MFI at 150° using the Eyring equation. The activation energies were similar for all zeolites with values between 140-150 kJ mol^{-1} (Figure S20, A, B). The calculations were done assuming an average activation energy of 145 kJ mol^{-1}.

2.4.1. Calculation of the distance between two hydronium ions neighbors

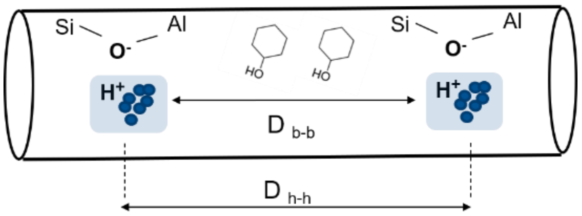

Scheme S2. MFI zeolite pore and the respective distances.

The mean distance between two hydronium ions neighbors (D_{h-h}) was estimated by the cube root of the medium zeolite total volume normalized to the number of $BAS_{(total)}$.[5]

Table S7. Parameters used for the calculation of the mean distance between two hydronium ions neighbors.

Samples	Unit cell volume (Å)^3	Density g cm^{-3}	Volume/N° H_3O^+ *10^{-21} (cm^3)	Volume/N° $H_3O^{+\ 1/3}$ (cm) *10^7	D_{h-h} (nm)
Al-MFI	5365.77	1.785	4.430	1.642	1.642
Al/Sn-8	5366.35	1.785	4.430	1.642	1.642
Al/Sn-4	5370.26	1.786	4.427	1.642	1.642
Al/Sn-2	5390.72	1.792	4.413	1.640	1.640
Al/Sn-1	5449.36	1.804	4.382	1.636	1.636

The mean distance between two hydronium ions neighbors free of water (D_{b-b}) was calculated according to the equation 12. The volume of hydronium ion was calculated considering 8, 10 and 12 waters, in sequence the diameter of this hydronium ion were estimated applying a cylinder model and a pore width of 0.55nm.[8]

$$D_{b-b} = D_{h-h} - D_{hydronium\ ion} \quad \textbf{(eq 12)}$$

Table S8. The mean distance between two hydronium ions neighbors free of water (D_{b-b}). The volume of hydronium ion was calculated considering 8, 10 and 12 waters.

Samples	D_{h-h} (nm)	D_{b-b} (8 water) (nm)	D_{b-b} (10 water) (nm)	D_{b-b} (12 water) (nm)
Al-MFI	1.642	1.205	1.096	0.987
Al/Sn-8	1.642	1.205	1.096	0.987
Al/Sn-4	1.642	1.204	1.095	0.987
Al/Sn-2	1.640	1.202	1.094	0.985
Al/Sn-1	1.636	1.199	1.090	0.981

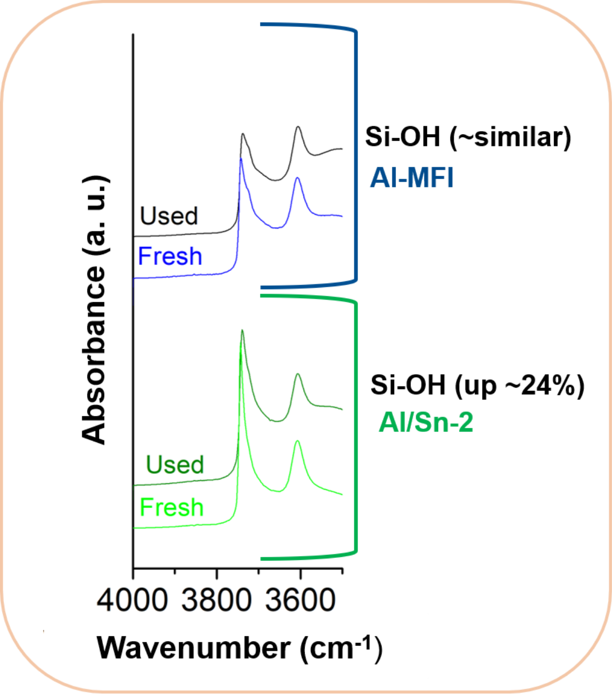

Figure S21. Comparison of the IR spectra at 4000-3500 cm^{-1} wavenumber range of the Al-MFI and Al/Sn-2 samples fresh and used in the reaction of dehydration of cyclohexanol at liquid phase (150°C; 20min; 40bar). Both samples were activated at 450°C for 1h on the syntheses MFI zeolites at 150°C.

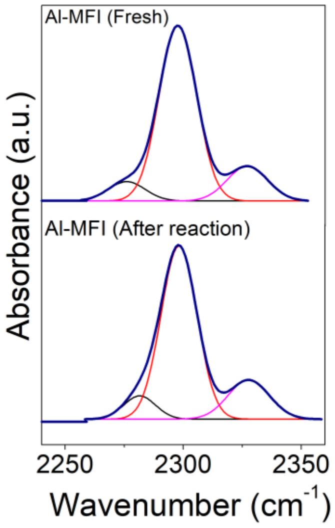

Figure S22. Comparison of the IR spectra after CD_3CN titration of the Al-MFI fresh and used in the reaction of dehydration of cyclohexanol at liquid phase (150°C; 20min; 40bar). Both samples were activated at 450°C for 1h followed by titration at 40°C. The deconvolution of the IR spectra was done with the ORIGIN software program using Gaussian-type curves; (black- Si-OH); (red- BAS_{Al}); (pink- Al extra framework).

Table S9. Acid site concentration is measured by the titration of CD_3CN.

Samples	Deuterated acetonitrile IR (mmol g^{-1})				
	$LAS_{(Sn)}$ (2308 cm^{-1})	$LAS_{(Sn)}$ (2316 cm^{-1})	$LAS_{(EFAL)}$ (2325 cm^{-1})	$BAS_{(Al)}$ (2296 cm^{-1})	$LAS_{(Sn)}$ total
Al/Sn-2 (fresh)	0.095	0.021	0.044	0.217	0.115
Al/Sn-2 (used)	0.040	0.064	0.040	0.220	0.104
Al-MFI (fresh)	-	-	0.031	0.211	-
Al-MFI (used)	-	-	0.036	0.200	-

3. References

(1) Topsøe, N. Y.; Pedersen, K.; Derouane, E. G. Infrared and Temperature-Programmed Desorption Study of the Acidic Properties of ZSM-5-Type Zeolites. *J. Catal.* **1981**, *70*, 41–52. https://doi.org/10.1016/0021-9517(81)90315-8.

(2) Harris, J. W.; Cordon, M. J.; Di Iorio, J. R.; Vega-Vila, J. C.; Ribeiro, F. H.; Gounder, R. Titration and Quantification of Open and Closed Lewis Acid Sites in Sn-Beta Zeolites that Catalyze Glucose Isomerization. *J. Catal.* **2016**, *335*, 141–154. https://doi.org/10.1016/j.jcat.2015.12.024.

(3) Pelmenschikov, A. G.; Van Santen, R. A.; Jänchen, J.; Meijer, E. CD_3CN as a Probe of Lewis and Bronsted Acidity of Zeolites. *J. Phys. Chem.* **1993**, *97*, 11071–11074. https://doi.org/10.1021/j100144a028.

(4) Jentys, A.; Warecka, G.; Derewinski, M.; Lercher, J. A. Adsorption of Water on ZSM5 Zeolites. *J. Phys. Chem.* **1989**, *93*, 4837–4843. https://doi.org/10.1021/j100349a032.

(5) Eckstein, S.; Hintermeier, P. H.; Zhao, R.; Baráth, E.; Shi, H.; Liu, Y.; Lercher, J. A. Influence of Hydronium Ions in Zeolites on Sorption. *Angew. Chem. Int. Ed.* **2019**, *58*, 3450–3455. https://doi.org/10.1002/anie.201812184.

(6) Yakimov, A. V.; Kolyagin, Y. G.; Tolborg, S.; Vennestrøm, P. N. R.; Ivanova, I. I. 119Sn MAS NMR Study of the Interaction of Probe Molecules with Sn-BEA: The Origin of Penta- and Hexacoordinated Tin Formation. *J. Phys. Chem. C* **2016**, *120*, 28083–28092. https://doi.org/10.1021/acs.jpcc.6b09999.

(7) Montejo-Valencia, B. D.; Salcedo-Pérez, J. L.; Curet-Arana, M. C. DFT Study of Closed and Open Sites of BEA, FAU, MFI, and BEC Zeolites Substituted with Tin and Titanium. *J. Phys. Chem. C* **2016**, *120*, 2176–2186. https://doi.org/10.1021/acs.jpcc.5b09815.

(8) Olson, D. H.; Kokotailo, G. T.; Lawton, S. L.; Meier, W. M. Crystal Structure and Structure-Related Properties of ZSM-5. *J. Phys. Chem.* **1981**, *85*, 2238–2243. https://doi.org/10.1021/j150615a020.
